# Supplementary material for: Energy-efficient CO2/CO interconversion by homogeneous copper-based molecular catalysts
Source: Nat Commun. 2023 Oct 27;14:6859. doi: 10.1038/s41467-023-42638-z (PMC10611766; doi:10.1038/s41467-023-42638-z)
Supplement: Supplementary file 1 — Supplementary Information [file 41467_2023_42638_MOESM1_ESM.pdf]

Supplementary Information

for

Energy-efficient CO_2/CO interconversion by homogeneous copper-based molecular catalysts

Somnath Guria[1], Dependu Dolui[1], Chandan Das[1], Santanu Ghorai[1], Vikram Vishal[2,3,4,5], Debabrata Maiti[1,3,4], Goutam Kumar Lahiri[1], Arnab Dutta[1,3,4,5]*

[1]Chemistry Department, Indian Institute of Technology Bombay, Powai, Mumbai 400076

[2]Earth Sciences Department, Indian Institute of Technology Bombay, Powai, Mumbai 400076

[3]Interdisciplinary Program Climate Studies, Indian Institute of Technology Bombay, Powai, Mumbai 400076

[4]National Center of Excellence CCU, Indian Institute of Technology Bombay, Powai, Mumbai 400076, India

[5]UrjanovaC Private Limited, Powai, Mumbai 400076, India

***Corresponding author: arnab.dutta@iitb.ac.in**

Table of Contents:

**Sr. No.**	**Detail Descriptions**	**Page No.**
**1. Experimental Section**		**4-9**
**1.1.**	Materials and Methods	**4-5**
**1.2.**	Synthetic Procedure	**5-6**
**1.3.**	Cyclic Voltammetry Study	**6-7**
**1.4.**	Bulk or Control Potential Experiment in aqueous buffer	**7**
**1.5.**	Spectroelectrochemistry (Optical)	**7**
**1.6.**	Rinse Test	**7-8**
**1.7.**	Chemical Catalysis Study	**8**
**1.8.**	GC Analysis	**8**
**1.9.**	Spectroelectrochemistry (FTIR)	**8**
**1.10.**	SCXRD Analysis	**9**
**1.11.**	Calculation for catalytic rate	**9**
**2. Results:**		**10-34**
**Figure S1.**	Optical spectra for complexes and ligands in DMF	**10**
**Figure S2.**	Comparative optical spectra for complexes in aqueous medium.	**10**
**Figure S3.**	Comparative FTIR spectra for complexes and ligands.	**11**
**Figure S4.**	Comparative EPR spectra.	**11**
**Figure S5.**	CV data for **C1** in DMF in Ar and scan rate dependence	**12**
**Figure S6-S7.**	Spectroelectrochemical UV-Vis data for **C1** in DMF.	**12-13**
**Figure S8.**	CV data for **C1** in DMF in CO_2 and scan rate dependence.	**14**
**Figure S9.**	Scan rate dependence for **C1** in DMF during CO oxidation.	**14**
**Figure S10.**	Comparative CV data for **C1** and **C2** with different holding time.	**15**
**Figure S11.**	Comparative CV data for **C1** with different amount of CO_2.	**15**
**Figure S12.**	Comparative reversible electrocatalytic responses by **C1** and **C2**	**16**
**Figure S13**	Background correction process.	**16**
**Figure S14.**	Comparative CV data for **C1** and **C2** with different amount of H_2O.	**17**
**Figure S15-S16.**	GC data for **C1** during CO_2 reduction and CO oxidation in DMF.	**17-18**
**Figure S17**	Bulk electrolysis of C1 complex in CO_2 atmosphere.	**18**
**Figure S18**	Comparative Faradic efficiency (FE) of **C1** and **C2** complexes.	**19**
**Figure S19**	Bulk electrolysis of **C1** and **C2** complexes in CO atmosphere.	**19**
**Figure S20-S21.**	GC data for **C1** during CO_2 reduction and CO oxidation in aqueous medium.	**20**
**Figure S22.**	CV data for **C2** in DMF in Ar and scan rate dependence	**21**
**Figure S23.**	Comparative CV data for complexes in DMF under Ar atmosphere.	**21**
**Figure S24.**	Comparative CV data for **C2** and TBAB in DMF.	**22**
**Figure S25.**	CV data for **C2** in DMF in CO_2 and scan rate dependence.	**22**
**Figure S26.**	GC data for **C2** during CO_2 reduction in DMF.	**23**
**Figure S27.**	Scan rate dependence for **C1** in DMF during CO oxidation.	**23**
**Figure S28.**	Comparative CV data for **C2** with different amount of CO_2.	**24**

**Figure S29.**	GC data for **C2** during CO oxidation in DMF.	**24**
**Figure S30**	Bulk electrolysis of **C2** complex in CO_2 atmosphere.	**25**
**Figure S31.**	Comparative CV data for **C2** in CO_2 with kinetic isotope effect.	**25**
**Figure S32-S33.**	GC data for **C2** during CO_2 reduction and CO oxidation in aqueous medium.	**26**
**Figure S34.**	The background corrected data for **C2** in aqueous solution.	**27**
**Figure S35.**	Rinse tests for **C1** and **C2**.	**27-28**
**Figure S36.**	The FE-SEM images.	**28**
**Figure S37.**	Optical spectra of pre and post electrolyzed solutions for **C1** and **C2**.	**29**
**Figure S38.**	Time dependent UV-Vis data for **C2** after addition of ascorbic acid under CO_2.	**29**
**Figure S39.**	Infrared spectroelectrochemical data for **C2** under CO_2.	**30**
**Figure S40-S41.**	GC data recorded for **C1-C2** during chemical catalysis under CO_2 atmosphere.	**31-31**
**Figure S42.**	Comparative CV data for **C1** after different time (day) interval.	**31**
**Figure S43-S44.**	GC data recorded for **C1-C2** during chemical catalysis under CO atmosphere.	**32**
**Figure S45.**	Comparative optical spectra for **C1** after adding CAN and water under CO atmosphere.	**33**
**Figure S46.**	Comparative FTIR spectra of **C1** under 100% CO_2 atmosphere	**33**
**Figure S47.**	Comparative CV data for the complexes under pure CO_2 and CO_2-CO mixture.	**34**
**Figure S48.**	HRMS data for the complexes recorded in methanol.	**34**
**Figure S49.**	^{1}H NMR spectrum data for **L2** recorded in $CDCl_3$.	**35**
**Table S1.**	Table for crystal data and refinement details for the complexes.	**35-36**
**References**		**36-37**

1. Experimental Section:

1.1. Materials and Methods: Copper perchlorate hexahydrate (ACS reagent grade, ≥ 99.0%), copper bromide, 2,6-diaminopyridine, and 2-amino pyridine was purchased from Sigma-Aldrich India. Nitrosobenzene, L-Ascobic acid, tetrabutylammonium tetrafluoroborate were purchased from TCI India Pvt. Ltd. Ceric ammonium nitrate was purchased from Merck India Pvt. Ltd. Acetone (HPLC and Spectroscopy), N, N-Dimethylformamide (HPLC and Spectroscopy), triethylamine (Dry, AR) and diethyl ether (AR), methanol (HPLC and gradient), chloroform (AR/ACS), triethanolamine (AR), hydrochloric acid (AR/ACS), sodium hydroxide (AR), sodium sulfate, anhydrous (AR/ACS), potassium chloride (AR), acetonitrile (HPLC and Gradient) and sodium chloride (AR) were purchased from Finar Chemicals Pvt. Ltd. India. All the analytical reagents were used as received without any further purification. Solvents are distilled using standard protocols. HPLC grade organic solvents and Millipore water (18.2 MΩ.cm resistivity at 298K) were used in all synthesis and chemical analysis. Glassware was oven dried before use. All the reactions were performed under N_2/Ar atmosphere unless otherwise stated. Nuclear Magnetic Resonance (NMR) Spectra were recorded at ~298K temperature using a Bruker Avance III Ascent FT spectrometer with working frequencies of 400 MHz for ^1H and 100 MHz for ^{13}C NMR. NMR signals are reported in δ (ppm) units while employing the solvent signals of $CDCl_3$ (δ=7.3ppm) and $(CD_3)_2SO$ (δ=2.50 ppm) as internal standard along with TMS (δ=0 ppm). The optical spectra were recorded on a PerkinElmer Lambda 1050 spectrometers using 1 cm path length in 2 mL volume Sterna make quartz cuvette. The FTIR spectra of pure solid samples were recorded on PerkinElmer (Spectrum-I) spectrometer using KBr pellets. HRMS of the samples was recorded with a Bruker maXis impact in positive mode. Cyclic voltammetry (CV) experiments were carried out at room temperature using Metrohm Autolab PGSTAT 204 potentiostat. All measurement was carried out either in dry DMF or in aqueous buffer medium, using 0.5 M $NaHCO_3$ in millipore water with a solute concentration of 1 mM in presence of anhydrous sodium sulphate (0.1 M) as supporting electrolyte. A standard three electrode system under N_2/Ar atmosphere was used with a 1 mm glassy carbon disc as a working electrode, Ag as a reference electrode connected by vycor tip and a platinum wire as counter electrode. All potentials are reported versus ferrocene ($Fc^{0/+}$) couple for organic medium, and hydroxymethyl ferrocene (FcOH) couple for aqueous medium ($FcOH^{+/0}$= +0.385 V vs. SHE), added in electrolyte at the end of each measurement. Electron paramagnetic resonance (EPR) experiments were performed in a Bruker EMX-Micro X-band EPR

spectrophotometer. The EPR data simulations were executed with EasySpin® software (version 5.2.35). Optical spectroelectrochemistry experiments were performed at room temperature in DMF solution using Ocean Optics spectrophotometer with 240.00 ms integration time and near 10 average scans. 2 ml of sample was placed in a 3.5 ml quartz cuvette (1 cm path length) placed in an external sample holder connected to light source and detector via optical fibres. System was further connected to a Metrohm Autolab PGSTAT204 using a 3 mm glassy carbon rod as working electrode, Pt wire as counter electrode, and Ag as a reference electrode with continuous Ar and CO_2 pressure respectively. Infrared spectroelectrochemistry experiments were performed employing an optically transparent thin–layer electrode (OTTLE) cell, equipped with Pt mesh as working electrode, Pt microwire as counter electrode, and Ag microwire as a pseudo–reference electrode. The electrodes of this cell was simulatenously connected to a Metrohm Autolab PGSTAT204 for electrochemical measurements. Bulk electrolysis setup includes a customized four-neck pear shape glass vessel (Volume 95 ml including head space), coiled 23 cm Pt wire as counter electrode in same chamber, Ag rod as a reference electrode, and reticulated vitreous carbon (1 cm x 2 cm) or glassy carbon rod (2π x 1.5 cm x 3 cm) electrode as working electrode. 14 ml of 1 mM complexes in DMF solutions or aqueous buffer ($NaHCO_3$, water) were used, respectively, during their chrono-coulometric experiments. pH of the aqueous solutions was measured in a bench top Labman LMPH-10 pH Meter (Scientific Instruments) before and after each experiment.

1.2. Synthetic Procedure:

1.2.1. 2-(phenylazo)pyridine (L1): The ligand 2-(phenyazo)pyridine (**L1**) was synthesized as per reported by Lahiri and co-workers.[1] In summary, 1.00 g 2-aminopyridine (10.60 mmol.) was added to a hot (60˚C) 50% aqueous NaOH solution (25 mL), followed by the addition of 3 mL of benzene. Next, 1.20 g of nitrosobenzene (11.20 mmol), dissolved in 10 mL of benzene, was added by a dropping funnel to the previous mixture over 30 minutes and warmed for 45 minutes. After extraction with benzene (3 x 100 mL), the organic solution was refluxed with charcoal for 2 hours, filtered, and concentrated under reduced pressure. The final product was separated by column chromatography, using alumina as stationary phase and 1-5% DCM-Hexane as mobile phase. Yield 1.5 g. (77.3%).

1.2.2. Bis-(2-(phenylazo)pyridine) copper(I) perchlorate (C1): C1 was synthesized following a modified version of the procedure reported by Datta *et al.*[2] Here, 974.5 mg of 2-

(phenylazo)pyridine (**L1**) (5.22 mmol) was added dropwise to a methanol/dichloromethane (1:2) blended solution (50 ml) containing 986.3 mg of $Cu(ClO_4)_2,6H_2O$ (2.66 mmol). The mixture was stirred overnight at room temperature, where a violet solution was obtained. This solution was evaporated under reduced pressure, and the residual solid was repetitively washed with n-pentane until the filtrate became colourless. Next, the violet colour sticky solid product was boiled in a 72ml methanol-water mixture (3:1). The resulting precipitate was filtered and dissolved in methanol for crystallization. Violet colour crystals appeared after 1 week that was further dried under vacuum. Yield: 1.13 g (80.3% with respect to **L1**). HRMS (ESI, +ve mode, MeOH) m/z for (M^+) [$C_{22}H_{18}Cu_1N_6$]: Calculated: 429.0883, Experimental: 429.0883 (**Figure S49A**). Optical spectral signals in DMF (λ_{max} in nm, ε in parentheses M^1 cm^{-1}): λ_{max}= 360 (25000); 580 (3929); 700 (907).

1.2.3. 6-amino-2(phenylazo)pyridine (L2): The ligand 6-amino-2(phenylazo)pyridine (**L2**) was synthesized from 2,6-diaminopyridine as follows. 2.00 g of 2,6-diaminopyridine (18.33 mmol) was dissolved in 15.0 mL of pyridine and mixed with 10.0 mL of 60% aqueous NaOH solution. Then 1.95 g of nitrosobenzene (18.2 mmol), dissolved in 10.0 mL pyridine, was added dropwise to the mixture for 3.0 hours, followed by 20.0 hours of reflux. The completion of the reaction was confirmed by thin-layer chromatography. The dark red mixture solution was diluted with water and extracted with dichloromethane (DCM) (3 x 100 mL). A dark red crude was obtained from evaporation of the organic layer, which was purified by column chromatography (100% DCM), using neutral alumina as stationary phase. Yield 378.0 mg (10.4% with respect to 2,6-diaminopyridine).

^1H NMR (400 MHz, δ in ppm, 298K, $CDCl_3$); δ= 7.99 (d, *J* = 7.9 Hz, 1H); 7.60 (t, *J* = 7.8 Hz, 1H); 7.49 (d, *J* = 7.49 Hz, 3H); 7.18 (d, *J* = 7.5 Hz, 1H); 6.59 (d, *J* = 8.1 Hz, 1H); 4.90 (s, 2H) (**Figure S50**).

1.2.4. Bis-(6-amino-2(phenylazo)pyridine) copper(I) Bromide (C2): The 6-amino-2-(phenylazo)pyridine (**L2**) (42.8 mg / 0.22 mmol) in 10 ml DCM was dropwise added (under N_2) to a methanol/dichloromethane (1:2) mixture containing 40.0 mg of $CuBr_2$ (0.11mmol). The mixture solution was stirred for 1 hour at room temperature. Finally, a green solution was obtained that was evaporated under the vacuum. Then the green solid remaining was boiled in a methanol-water mixture. The precipitate from the mixture was filtered and dissolved in methanol and

recrystallized in methanol with diffusing n-hexane. Small green crystals appeared in a week that was dried under vacuum. Yield 15.0 mg (25% with respect to **L2**). HRMS (ESI, +ve mode, MeOH) m/z for (M^+) [$C_{22}H_{20}Cu_1N_8$]: Calculated: 459.1101, Experimental: 459.1101 (**Figure S49B**). Optical spectral signals in DMF (λ_{max} in nm, ε in parentheses M^1 cm^{-1}): λ_{max}= 425 (26500); 601 (5860); 739 (1233).

1.3. Cyclic voltammetry study in organic solvent and in aqueous pH buffer: The cyclic voltammetry experiment was primarily executed in an organic medium (dry DMF). Additionally, a few experiments were performed in an aqueous solution (0.5 M $NaHCO_3$ buffered medium, pH 6.5). The analyte complex concentration was maintained at ~1 mM in all the cases unless mentioned otherwise. In an organic medium, 0.1 M tetrabutylammonium tetrafluoroborate (TBAF, $nBu_4N^+BF_4^-$) was employed as the supporting electrolyte. In water, Na_2SO_4 was added for the same role. The cyclic voltammograms were recorded using a typical three-electrode assembly, containing a 1 mm diameter glassy carbon disc working electrode, Pt-wire counter electrode, and Ag/AgCl (saturated KCl) reference electrodes. The applied potential values during the experiments were internally standardized either by using ferrocene (in organic medium) or hydroxymethyl ferrocene (FcOH). Hence, all the potential values in organic media in this study were reported against ferrocene couple ($Fc^{+/0}$), while the same in aqueous media was done against RHE by referencing hydroxymethyl ferrocene couple ($FcOH^{+/0}$ ~ +0.385 V).

1.4. Bulk or Control Potential Experiment in aqueous buffer: Bulk Electrolysis (BE) or control potential experiment (CPE) was performed in an air-tight 95 ml four neck glass vessel. Three of these outlets were fitted with various electrodes; 2 cm x 1 cm vitreous carbon as a working electrode; 23 cm coiled platinum wire as a counter electrode, and Ag wire as a reference electrode. The last outlet was closed by a B-14/20 suba® seal rubber septum, which was used for purging CO_2 or CO or Ar (for 30 minutes) before the experiments and for headspace gas collection. During an experiment, 14 ml of 1 mM complexes were added to the vessel, all electrodes (along with a magnetic bead) were inserted along with a B-14/20 rubber septum cap (in a gas-tight manner). Then, the chrono-coulometric experiment was started at corresponding catalytic potentials in both organic and aqueous media. The reaction solution was continuously stirred during the experiment. Headspace gas was collected by a GASTIGHT® PTFE leur-lock 1000 series (1001TLL) 1 ml

Hamilton® syringe after certain time intervals, and it was analyzed via gas chromatography (GC) instrument on TCD/FID mode.

1.5. Spectroelectrochemistry (Optical): Optical spectroelectrochemistry experiments were performed via an Ocean Optics spectrophotometer in tandem with a Metrohm Autolab PGSTAT204 potentiostat. The sample was placed in a 3.5 ml quartz cuvette (1 cm path length) fixed in an external sample holder and connected to a light source and a detector via optical fibres. The cuvette was also fitted with a 3 mm glassy carbon rod working electrode, a Pt wire counter electrode, and a silver wire reference electrode. A controlled potential electrolysis (CPE) experiment was performed with the use of the potentiostat, while the respective changes in the optical spectrum were monitored with the spectrophotometer.

1.6. Rinse Test: A rinse test for complexes has been carried out in an organic medium (DMF) to probe the homogeneous or heterogeneous nature of the catalysis. For this purpose, we have executed three consecutive runs as follows. A complete CV was recorded for the complexes in the corresponding organic medium under the CO_2 atmosphere in the first run. Then the working electrode was thoroughly rinsed with water. Then this electrode was properly polished with 0.25 µm alumina powder. Afterward, a second run was performed with the same complex solution with the cleaned working electrode. However, this second run was stopped at the potential where the maximum CO_2 reduction signal was observed. Then working electrode was only rinsed (without any polishing) with water, and a third cyclic voltammogram was recorded in a different solution that contained only blank DMF and the electrolyte but no complex. This third scan was initiated again from the maximum catalytic response potential observed for CO_2 reduction. The absence of any significant catalytic response in the third scan in the reduction direction validates the homogeneous CO_2 reduction mechanism.

1.7. Chemical Catalysis: ~1 mM stock solutions for each complex were prepared in DMF. From that stock solution, 5mL was taken in a 105 mL Schlenk tube connected to a magnetic rotor. Next, a stream of CO_2 was purged into the solution for 30 minutes along with stirring to prepare a CO_2-saturated solution. Then varying amounts of (0-10 mM) ascorbic acid were added to this Schlenk tube under anaerobic conditions while stirring at 500 rpm for 22h. During this experiment, 0.5 ml headspace gas was taken out from the Schlenk tube and injected into the GC for analysis at various time intervals. A similar procedure was adopted for CO oxidation with the sample under CO-

saturated conditions. Here, cerium ammonium nitrate (CAN) and 6.1 M water were added instead of ascorbic acid to regenerate Cu(II) species. A number of control experiments were performed in the presence of ascorbic acid or CAN under CO_2 and CO atmosphere, respectively, in blank DMF.

1.8. Gas chromatography analysis: The amount of CO_2/CO evolved during catalysis were quantified by using Dhruva CIC gas chromatography (GC) instrument with TCD/FID detector with a 5 Å molecular sieve/Porapak at room temperature. Instrument calibration build up curve was created manually by injecting variable amount (0.5%-2%) of known CO_2 and CO gas mixtures.

1.9. Spectroelectrochemistry (FTIR): An optically transparent thin–layer electrode (OTTLE) cell, equipped with a Pt mesh working electrode, a Pt microwire counter electrode, and Ag microwire pseudo–reference electrode were employed for this study along with a Metrohm Autolab PGSTAT204. All the FTIR spectra were measured using a Perkin–Elmer FTIR spectrometer set in absorbance mode. All the measurements were carried out at room temperature with a ~30mM complex concentration. Data was also recorded for a CO_2-saturated blank DMF solution, subtracted from all the experimental data collected under the CO_2 atmosphere.

1.10. Single crystal X-ray diffraction study: Crystals of **C1** and **C2** complexes were grown from methanol/Hexane layering by a solvent evaporation method. Suitable crystals for each complex were selected and mounted on a cryo-loop using cryoprotectant paratone oil. Single crystal diffraction data for both complexes were collected at 150K and 100K on a Bruker D8 Quest diffractometer equipped with an Incoatec Microfocus Source (IμS 3.0 Mo Kα, $\lambda = 0.71073$ Å) and a PHOTON II detector. X-ray diffraction intensities were collected, integrated and scaled with APEX4 software. Empirical absorption correction was applied to the data by employing multi-scan method with SADABS programming.[3] Structure was solved by intrinsic phasing with SHELXT[4] and, refined by full-matrix least-square methods on F^2 using SHELXL using the ShelXle along with Olex2 interface.[5,6] All non-hydrogen atoms were refined with anisotropic displacement parameters. The hydrogen atoms were introduced at a calculated positions and were treated as riding atoms with an isotropic displacement parameter, C-H = 0.93-0.98 A° with Uiso(H) = 1.5UeqI for methyl groups, Uiso(H) = 1.2Ueq(C, N) for all other C—H and N—H bonds and O—H = 0.82 A˚ [Uiso(H) = 1.5Ueq(O)]. Mercury, PLATON and publCIF was used for molecular graphics, validation and to prepare material for publication.[7–9] Details of crystal data collections

and data refinement parameters are given in Table **S1**. The complete crystallographic information file (CIF) for **C2** complex was deposited in Cambridge crystallographic data centre (**CCDC 2122626**)

1.11. Calculation of catalytic rate: Catalytic rate of the complexes was measured from this equation S1:

$$\frac{i_{cat}}{i_p} = \frac{n}{0.4463}\sqrt{\frac{RTk_{obs}}{Fv}}$$ **(Equation S1)**

where, i_{cat} = catalytic current, i_p = stoichiometric current, n = number of electrons involved in this process, R = universal gas constant, T = temperature in K, F = 1 Faraday, and ν = scan rate.

2. Results:

2.1. Supplementary Figures:

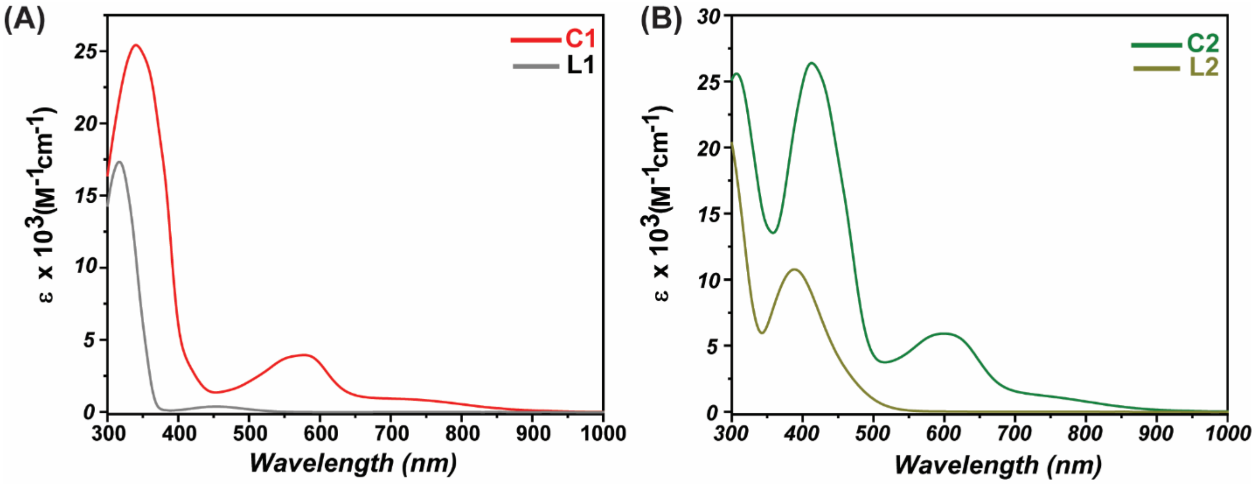

Figure S1. Optical spectra data for the copper complexes and corresponding ligands in DMF. (**A**) Comparative spectra of **L1** (grey trace) and **C1** (red trace), **(B)** Comparative spectra of **L2** (dark yellow trace), and **C2** (green trace). All the spectra were recorded at room temperature scanning in the 300-1000 nm range.

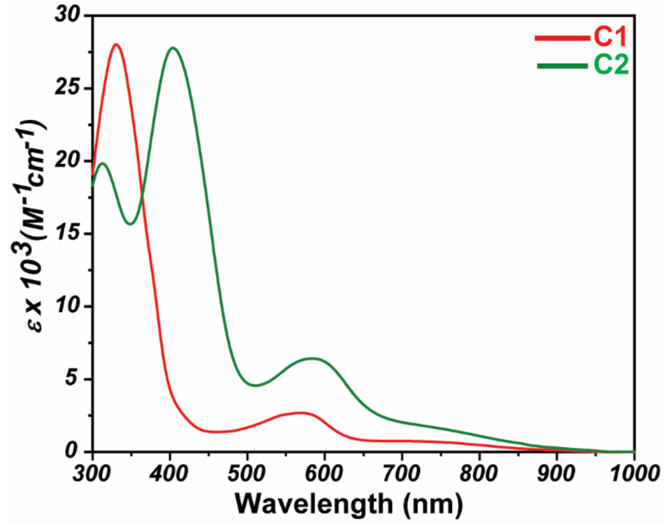

Figure S2. Comparative optical spectra of the **C1** (red trace); **C2** (green trace) complexes; recorded in aqueous media (pH 6.5, $NaHCO_3$ buffer). All the spectra were recorded at room temperature scanning in the 300-1000 nm range.

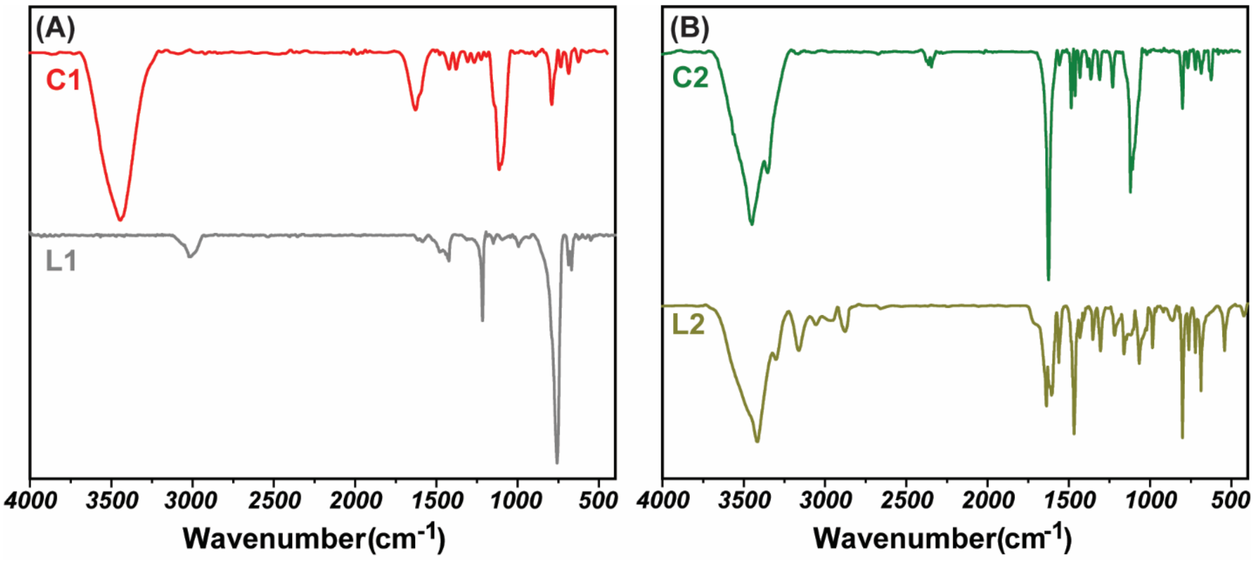

Figure S3. FTIR spectrum comparison between: (A) **C1** (red trace), and **L1** (grey trace); (B) **C2** (olive trace), and **L2** (dark green trace). Spectrum were recorded in % transmittance mode at room temperature using solid KBr pellet from 4000-400 cm^{-1} range.

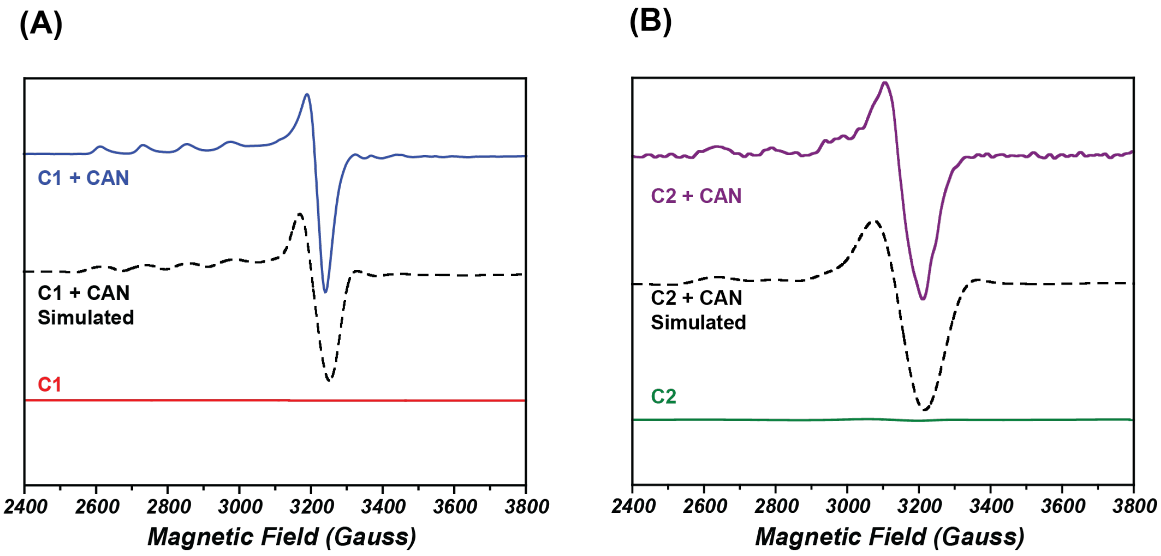

Figure S4. Comparative EPR spectrum of (**A**) **C1** complex before treating with CAN (cerium ammonium nitrate) (solid red trace), and after treating with equivalent amount of CAN (solid blue trace) along with the simulated data (dotted black trace); (**B**) **C2** complex before treating with CAN (solid green trace), and after treating with equivalent amount of CAN (solid violet trace) along with the simulated data (dotted black trace). Data were recorded in DMF at 100K temperature. The spin Hamiltonian parameters were obtained by simulating the experimental spectrum of the samples with EasySpin® software.

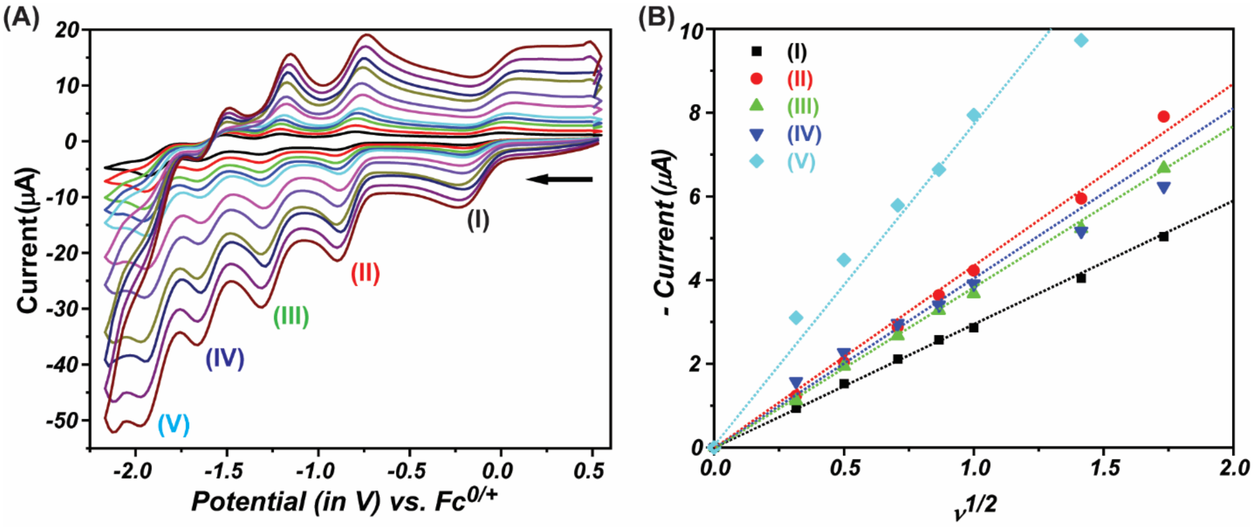

Figure S5. Cyclic Voltammetry (CV) study in Ar atmosphere for **C1** complex; (**A)** CV with different scan rate (0.1 V/sec to 10 V/sec) ; (B) $\nu^{1/2}$ vs reductive current plots of each positions. Data were recorded in DMF at 289K temperature. Arrow describe origin and direction of scan.

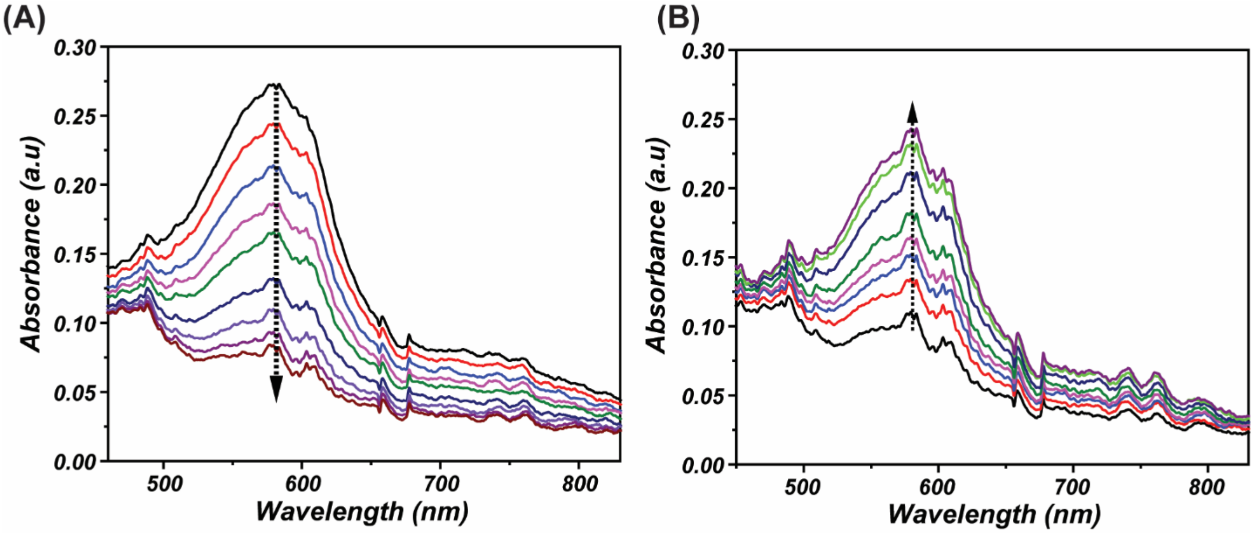

Figure S6. The gradual change in the optical spectra of 5 x 10^{-5} M **C1** complex under the application of (**A**) +0.06 V (vs. $Fc^{+/0}$) and (**B**) -0.20 V (vs. $Fc^{+/0}$) recorded over the period of 30 minutes during a spectroelectrochemical experiment. All data were recorded in DMF media at 298K temperature.

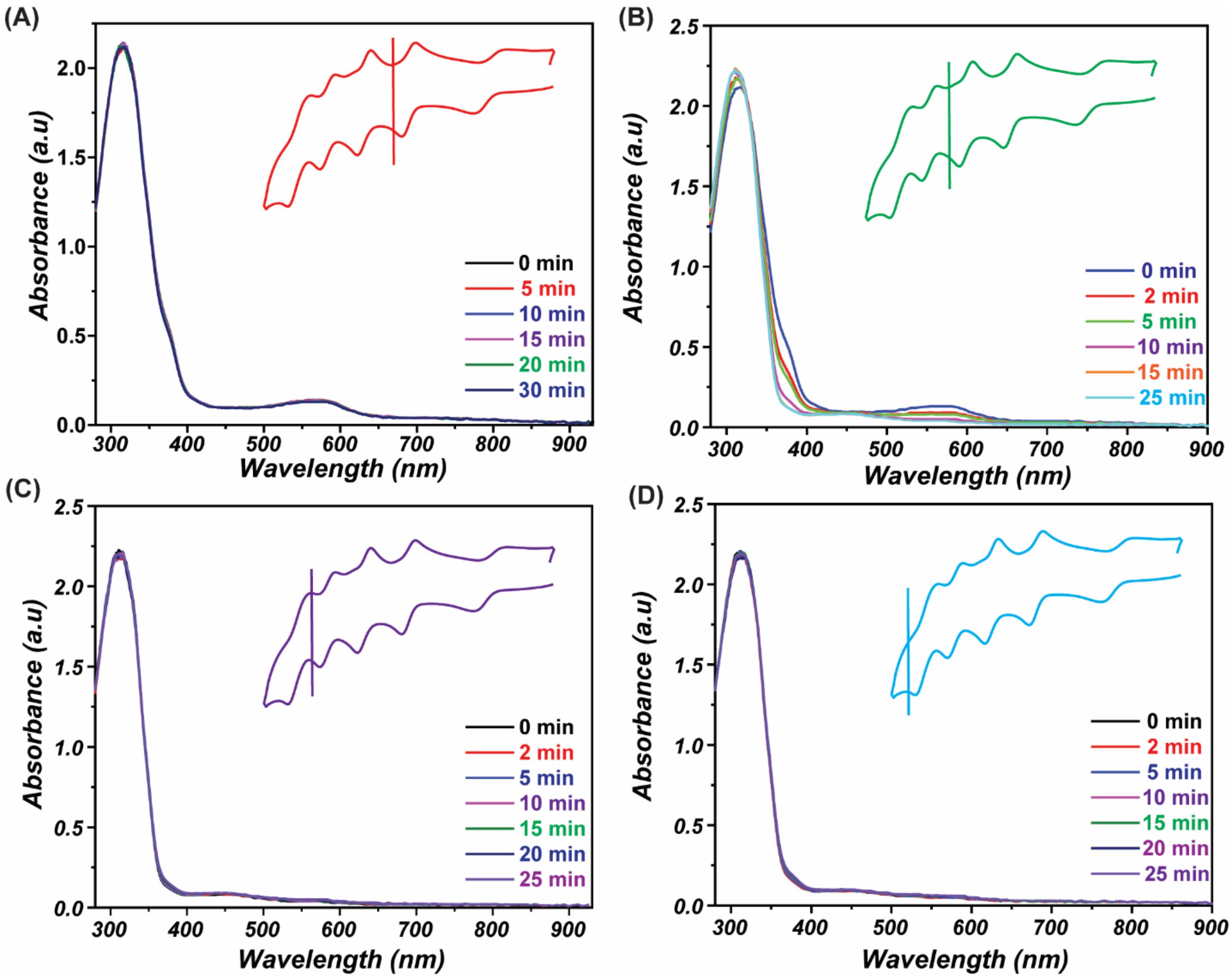

Figure S7. The changes in the optical spectra for 5 x 10^{-5} M **C1** complex during a spectroelectrochemistry experiment executed at (**A**) -1.15 V (vs. $Fc^{+/0}$), (**B**) -1.55 V (vs. $Fc^{+/0}$), (C) -1.80 V (vs. $Fc^{+/0}$), and (D) -2.05 V (vs. $Fc^{+/0}$). The position of the applied potential on the corresponding CV during the spectroelectrochemical experiment are showcased in the inset for each figure. All data were recorded in DMF media at 298K temperature.

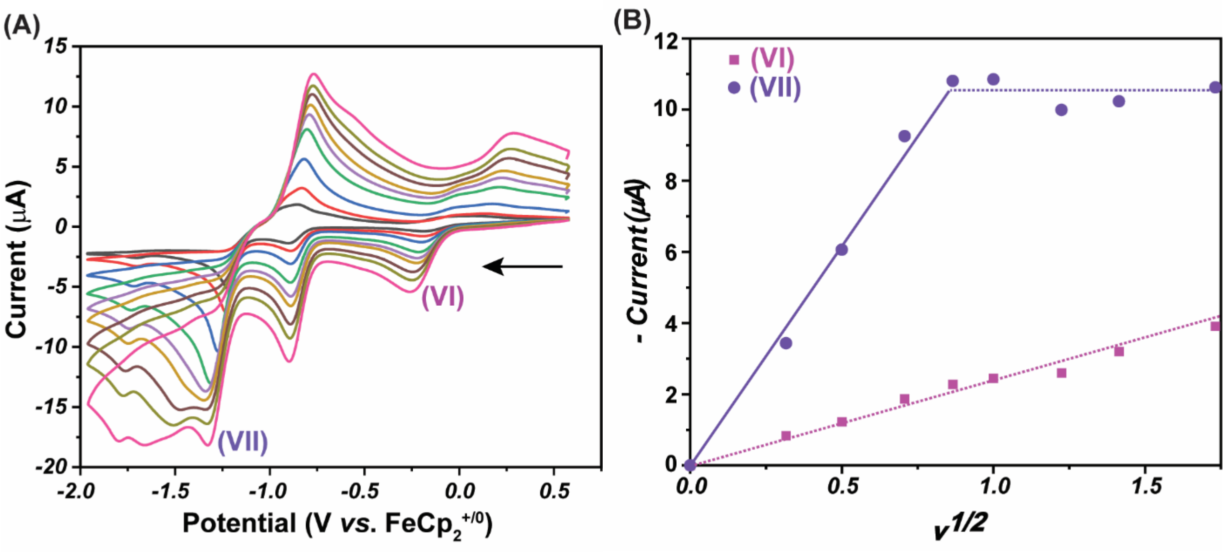

Figure S8. (**A**) The cyclic voltammograms recorded for **C1** under CO_2 atmosphere at variable scan rates (0.1 V/sec to 3.0 V/sec). (**B**) The comparative trend of change in reduction current vs. square root of scan rate ($\nu^{1/2}$) for the Cu(II/I) (violet trace) and the CO_2 reduction signal (purple trace). All data were recorded in DMF at 298K temperature. The horizontal arrow describes the initial scan direction.

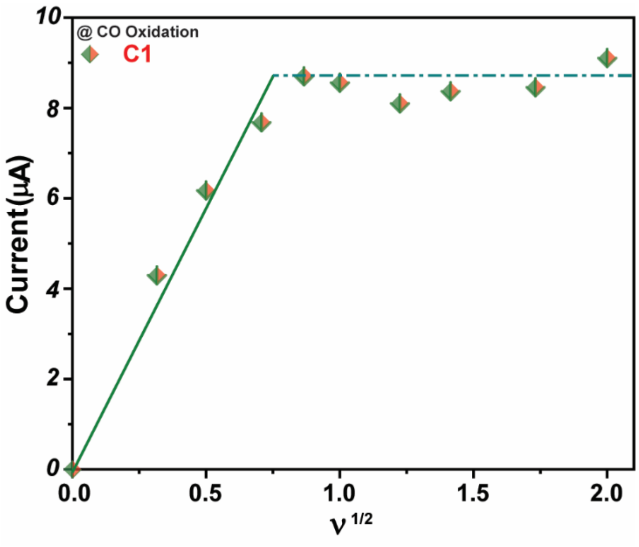

Figure S9. The change in oxidation current vs. square root of scan rate ($\nu^{1/2}$) observed for CO oxidation signal (green-orange diamonds) for **C1**. All data were recorded in DMF at 289K temperature.

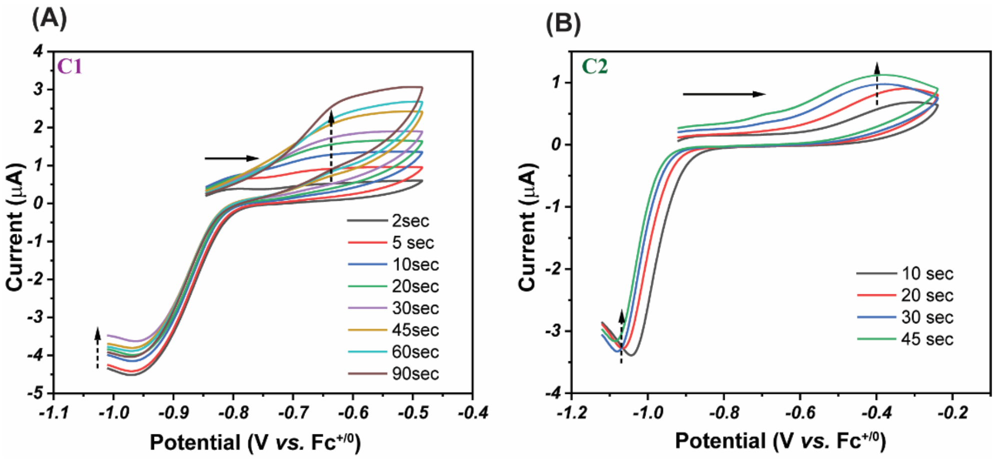

Figure S10. The comparative cyclic voltammograms recorded for **C1** and **C2** at variable holding times. **(A)** Holding potential for **C1** was -1.0 V vs $Fc^{+/0}$ under saturated CO_2 atmosphere and data were recorded in 9M H_2O/DMF at 289K temperature with a scan rate of 0.05 V/s. **(B)** Holding potential for **C2** was -1.01 V vs $Fc^{+/0}$ under saturated CO_2 atmosphere and data were recorded in 0.8M H_2O/DMF at 289K temperature with a scan rate of 0.05 V/s The horizontal arrow depicts the initial scan direction. The dotted vertical arrow shows the change in CO_2 reduction and CO oxidation signal.

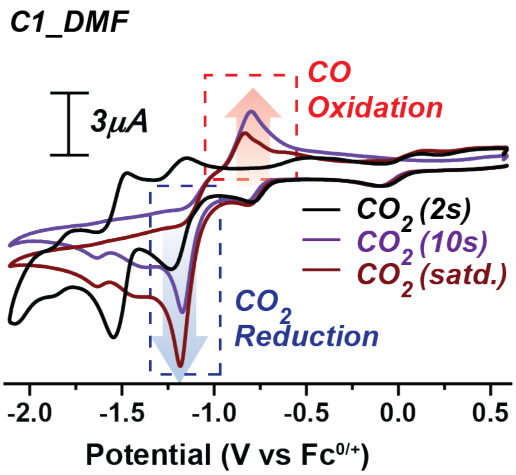

Figure S11. The cyclic voltammograms recorded for **C1** complex in DMF under variable CO_2 purging time: 2 seconds (black trace), 10 seconds (violet trace), and saturated CO_2 (brown trace); in DMF solution. The blue and red arrow showcase the changes in CO_2 reduction and CO oxidation signatures, respectively. All the data were recorded at 0.1 V/s scan rate in DMF media at 298 K.

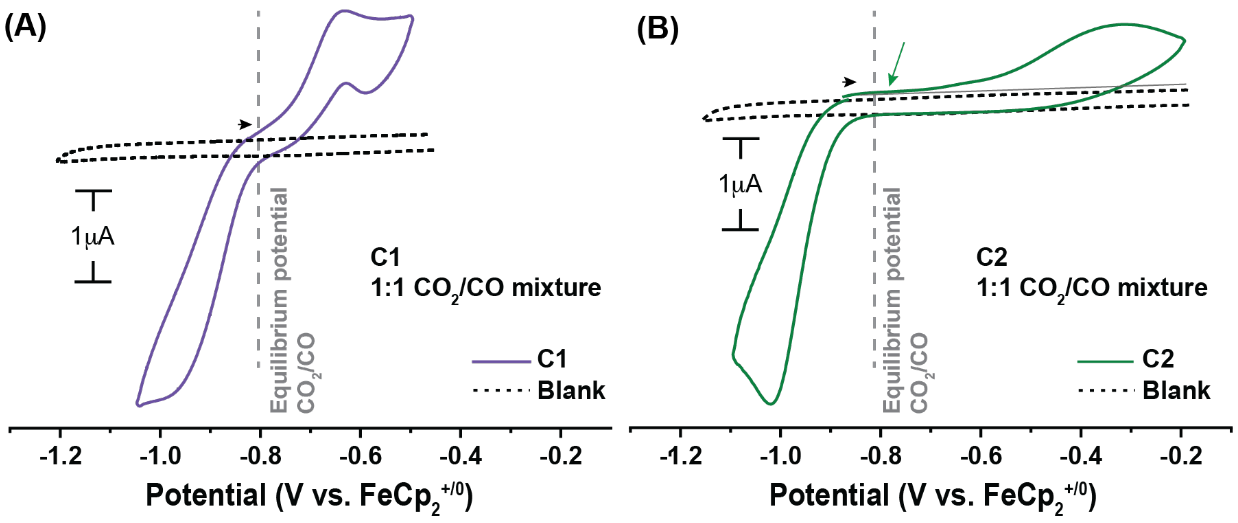

Figure S12. The comparative reversible electrocatalytic CO_2 reduction/CO oxidation behavior exhibited by (**A**) **C1** (blue trace) and (**B**) **C2** (green trace) recorded under 1 atm CO_2. This experiment was performed by starting from the CO_2/CO equilibrium potential (i.e., the potential around which the CO_2 reduction or CO oxidation takes place in either direction, depicted by the vertical dotted black line) while holding the potential at maximum CO_2 reduction signal for 20 s. Initially the potential was applied in the anodic direction as showcased by the horizontal black arrow. All the data were recorded in DMF at 298 K with a scan rate of 0.1 V/s.

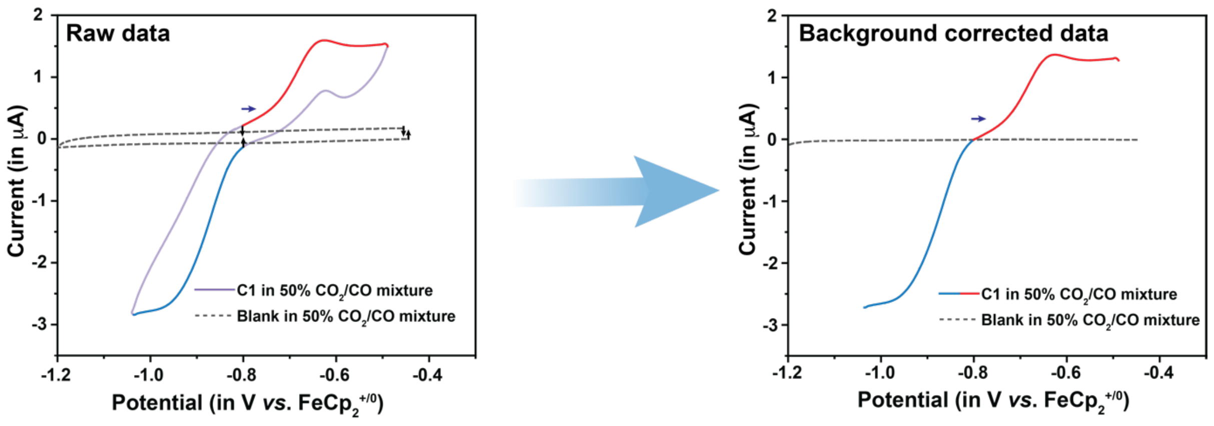

Figure S13. The background correction process to convert the raw data to highlight CO_2 reduction and CO oxidation signatures.

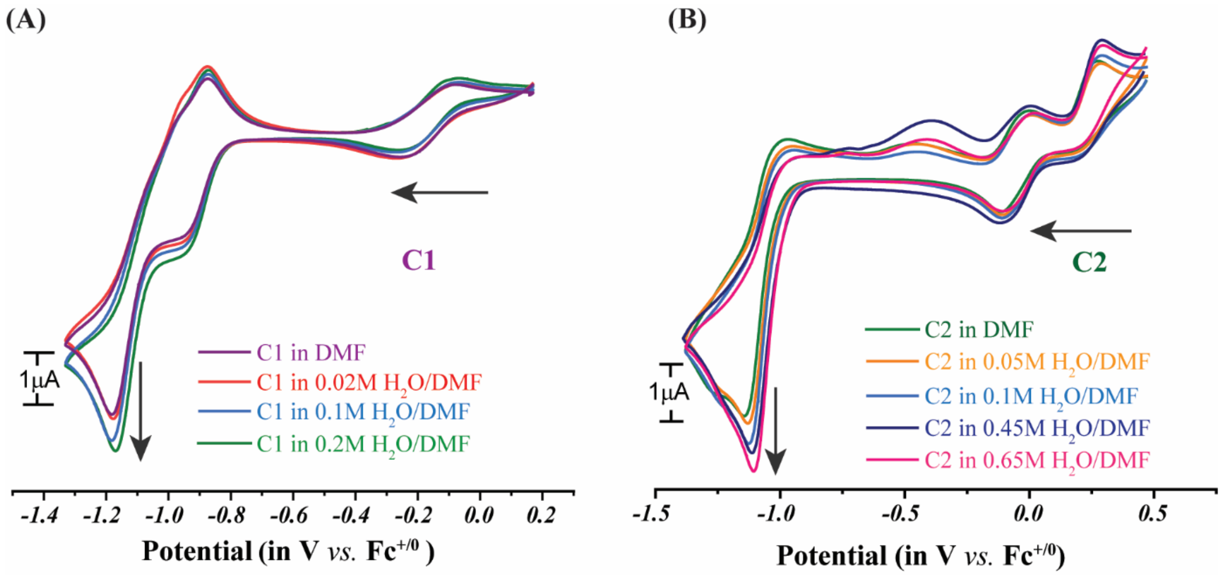

Figure S14. The comparative cyclic voltammograms of **C1** and **C2** under 1 atm CO_2 (black trace) in DMF. The violet trace displays the data collected for the same sample in the presence of 6.1 M water under 1 atm CO_2 in DMF. All data were collected at 0.05 V/s scan rate. The horizontal arrows depict the initial scan direction.

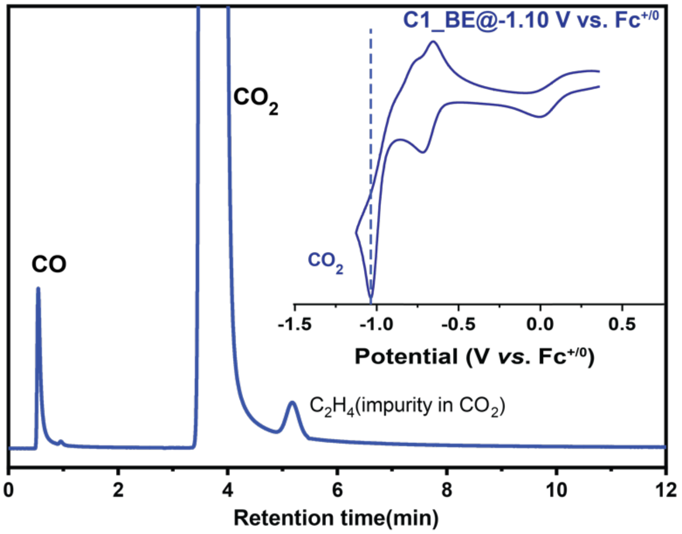

Figure S15. The gas chromatography (GC) data recorded for the head-space gas analyte generated during the bulk electrolysis of **C1** performed at -1.10 V vs. $Fc^{+/0}$ in DMF under 1 atm CO_2. The inset includes the cyclic voltammogram of **C1** indicating the precise position of the applied potential during the bulk electrolysis.

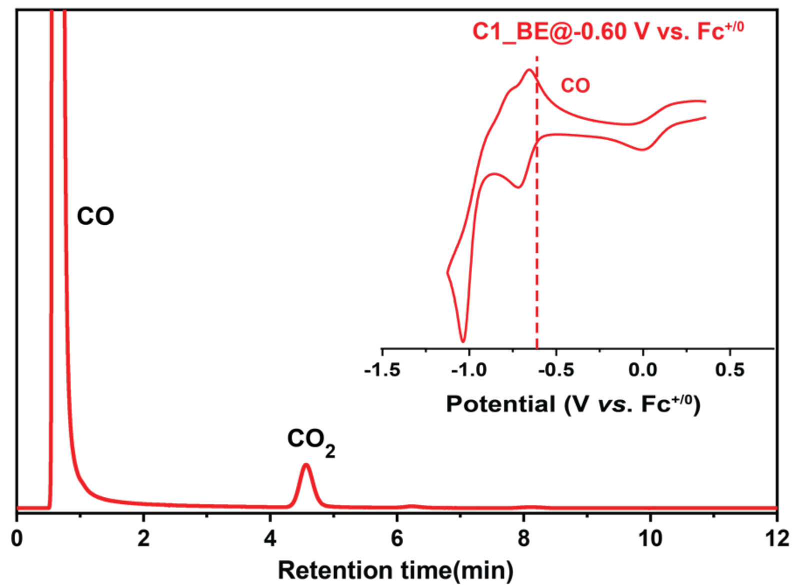

Figure S16. The gas chromatography (GC) data recorded for the head-space gas analyte generated during the bulk electrolysis of **C1** performed at -0.60 V vs. $Fc^{+/0}$ in DMF under 1 atm CO. The inset includes the cyclic voltammogram of **C1** indicating the precise position of the applied potential during the bulk electrolysis.

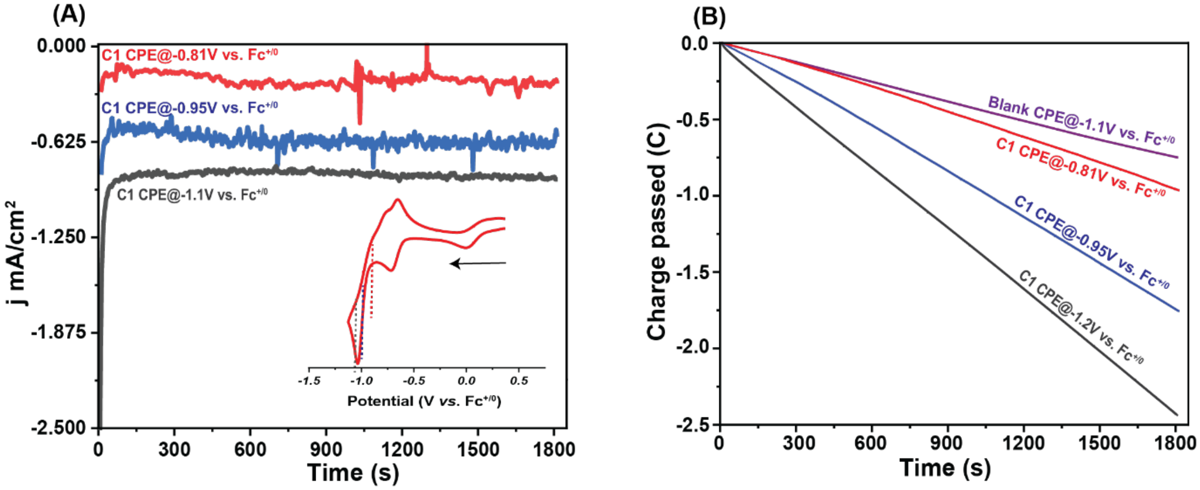

Figure S17. Bulk electrolysis of **C1** complex in CO_2 atmosphere. **(A)** Current *vs*. time plots at variable potentials, and **(B)** Corresponding charge passed *vs*. time plots at variable applied potential values.

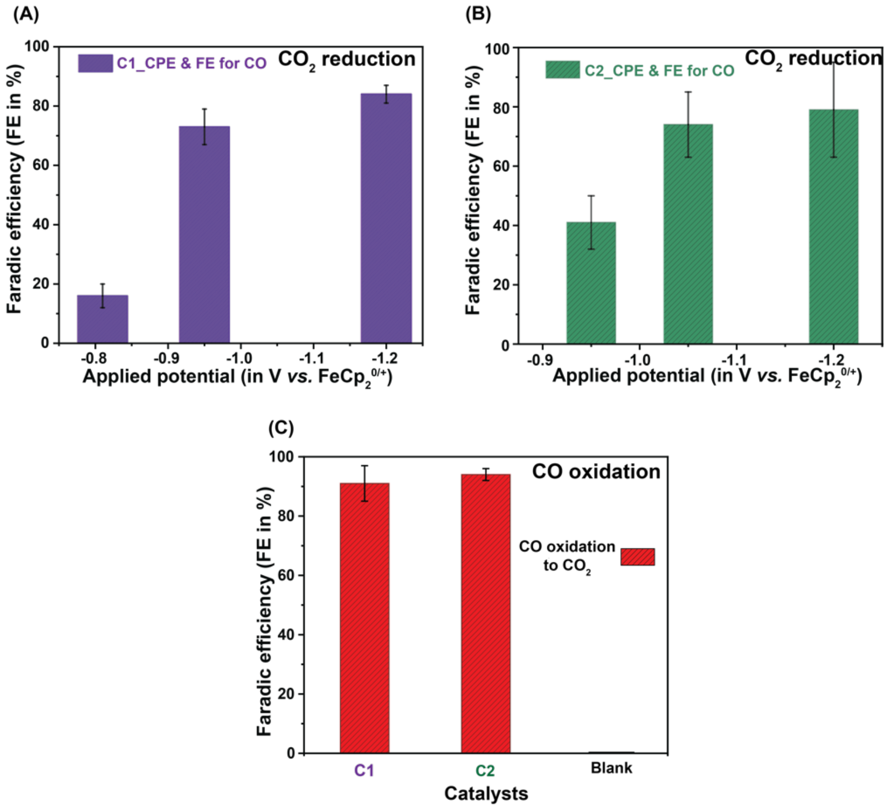

Figure S18. Comparative Faradic efficiency (FE) of **C1** and **C2** complexes for each bulk electrolysis experiment. (A) FE for CO formation by **C1** complex (B) FE for CO formation by **C2** complex. (C) FE for CO_2 formation by **C1** and **C2** complexes.

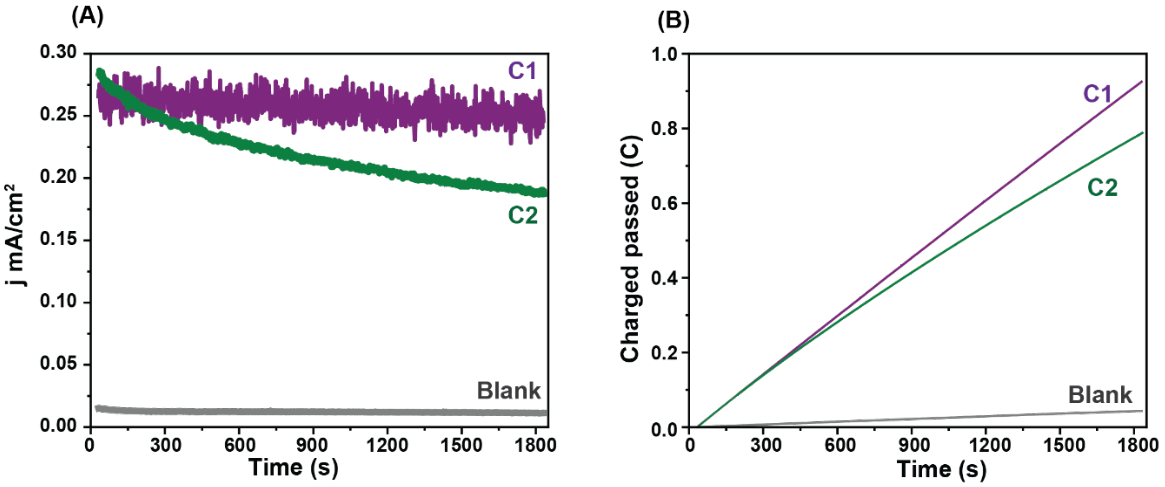

Figure S19. Bulk electrolysis of **C1** and **C2** complexes in CO atmosphere. (A) Current *vs*. time plots (B) Corresponding charge passed *vs*. time plots.

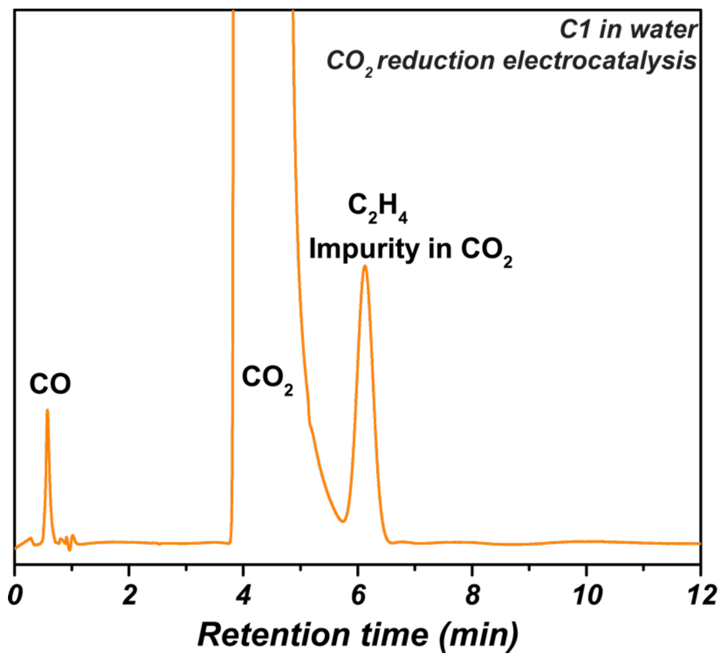

Figure S20. The gas chromatography (GC) data recorded for the head-space gas analyte generated during the bulk electrolysis of **C1** performed at -0.65 V vs. RHE in aqueous media (pH 6.5) under 1 atm CO_2.

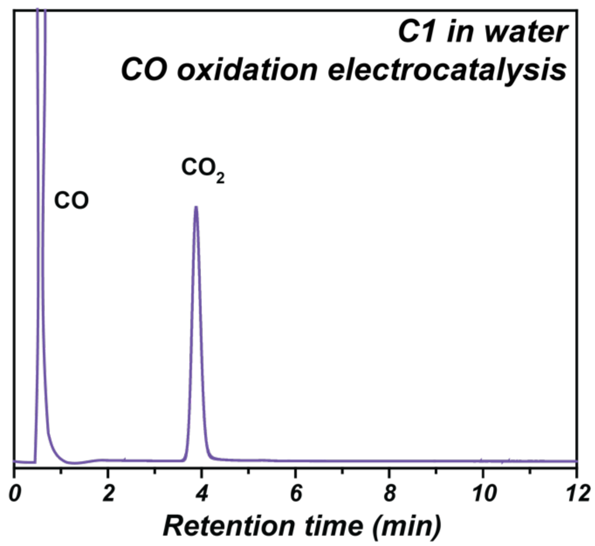

Figure S21. The gas chromatography (GC) data recorded for the head-space gas analyte generated during the bulk electrolysis of **C1** performed at -0.45 V vs. RHE in aqueous media (pH 6.5) under 1 atm CO.

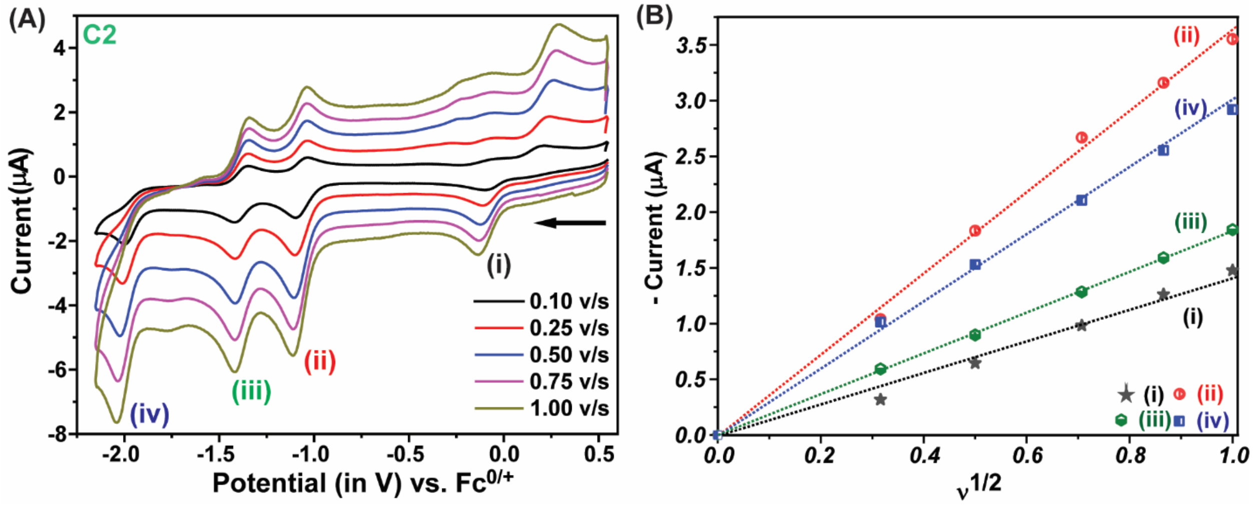

Figure S22. Cyclic Voltammetry (CV) study in Ar atmosphere for **C2** complex; (**A)** CV with different scan rate (0.1 V/sec to 1 V/sec) ; (B) $\nu^{1/2}$ vs reductive current plots of each positions. Data were recorded in DMF at 289K temperature. The horizontal arrow depicts the initial scan direction.

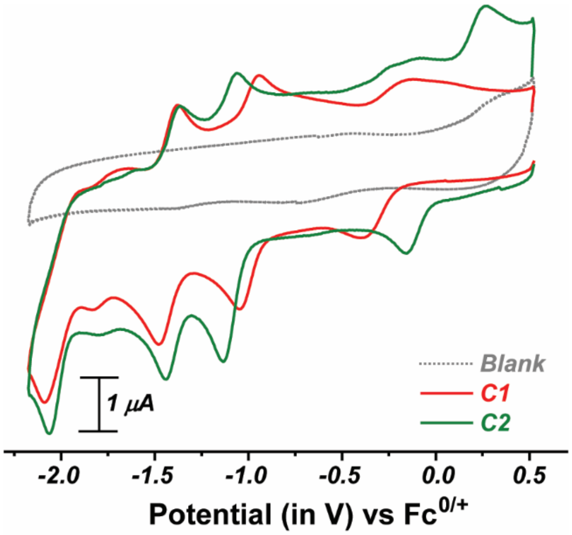

Figure S23. Comparative cyclic voltammograms recorded for blank (grey dotted trace), **C1** (solid red trace), and **C2** (solid green trace) under Ar atmosphere. Data were recorded in DMF media at 289K temperature with 0.1 V/s scan rate.

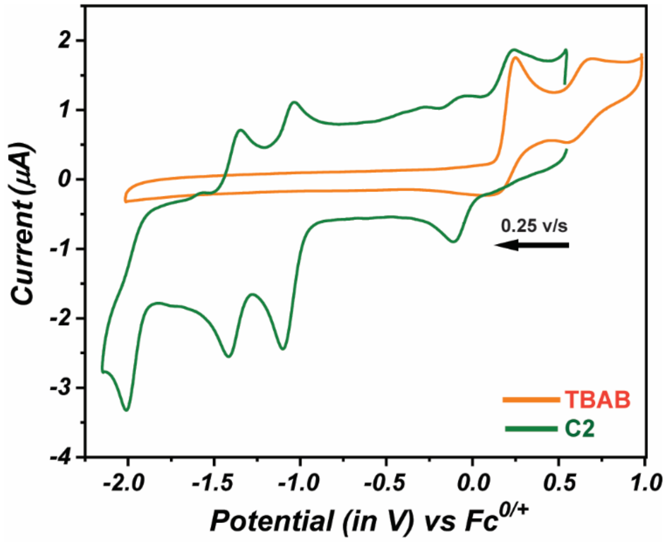

Figure S24. Comparative cyclic voltammograms recorded for **C2** complex (green trace) and Tetrabutylammonium bromide (TBAB) (orange trace) under Ar atmosphere in DMF. All data were recorded in DMF at 298K. The horizontal arrow describes the initial direction of the scan.

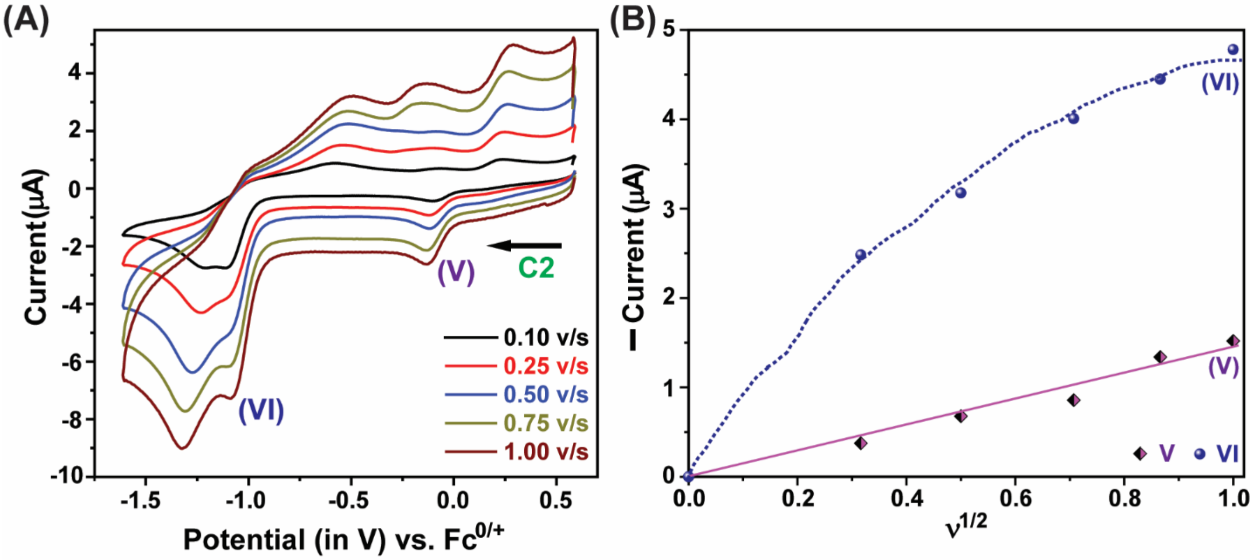

Figure S25. (**A**) The cyclic voltammograms recorded for **C2** under CO_2 atmosphere at variable scan rates (0.1 V/sec to 1.0 V/sec). (**B**) The comparative trend of change in reduction current vs. square root of scan rate ($\nu^{1/2}$) for the Cu(II/I) (violet trace) and the CO_2 reduction signal (blue trace). All data were recorded in DMF at 298K temperature. The horizontal arrow describes the initial scan direction.

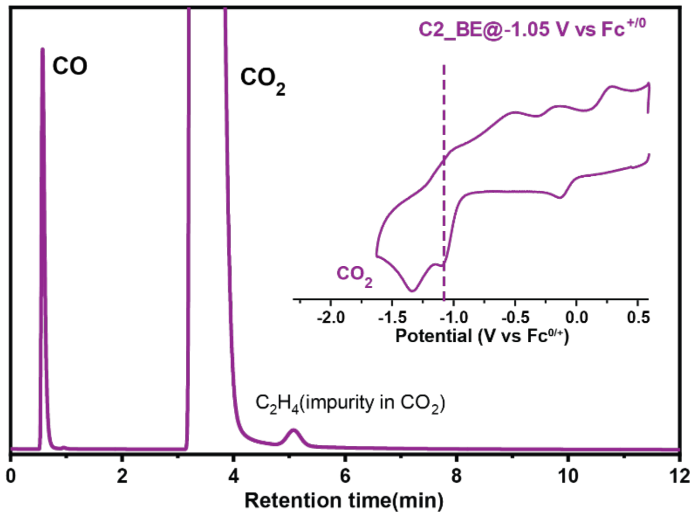

Figure S26. The gas chromatography (GC) data recorded for the head-space gas analyte generated during the bulk electrolysis of **C2** performed at -1.05 V vs. $Fc^{+/0}$ in DMF under 1 atm CO_2. The inset includes the cyclic voltammogram of **C2** indicating the precise position of the applied potential during the bulk electrolysis.

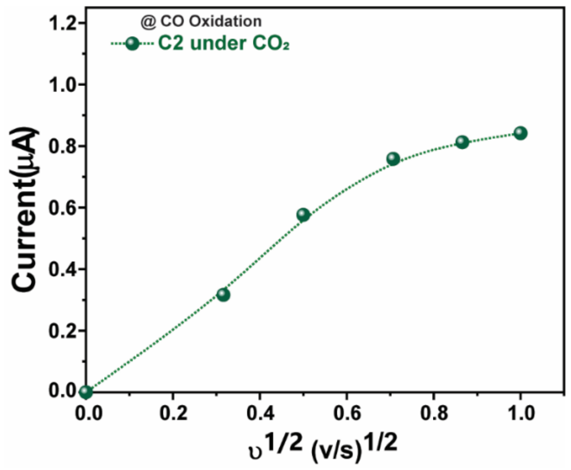

Figure S27. The change in oxidation current vs. square root of scan rate ($\nu^{1/2}$) observed for CO oxidation signal (green spheres) for **C2**. All data were recorded in DMF at 289K temperature.

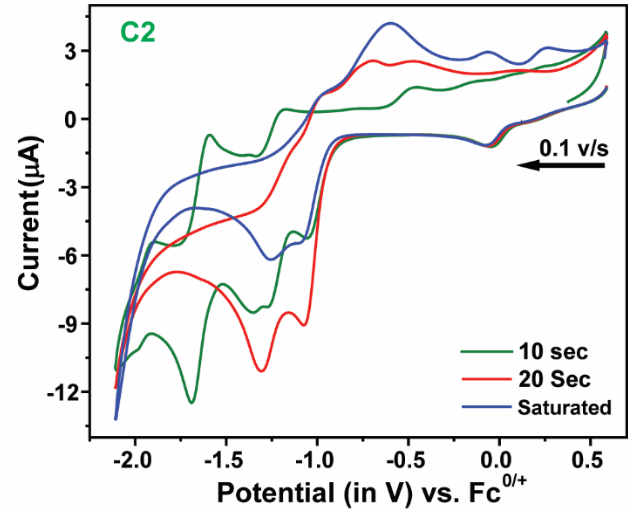

Figure S28. The cyclic voltammograms recorded for **C2** complex in DMF under variable CO_2 purging time: 10 seconds (green trace), 20 seconds (red trace), and saturated CO_2 (blue trace); in DMF solution. All data were recorded at 0.1 V/s scan rate in DMF media at 298 K. The horizontal arrow depicts the initial scan direction.

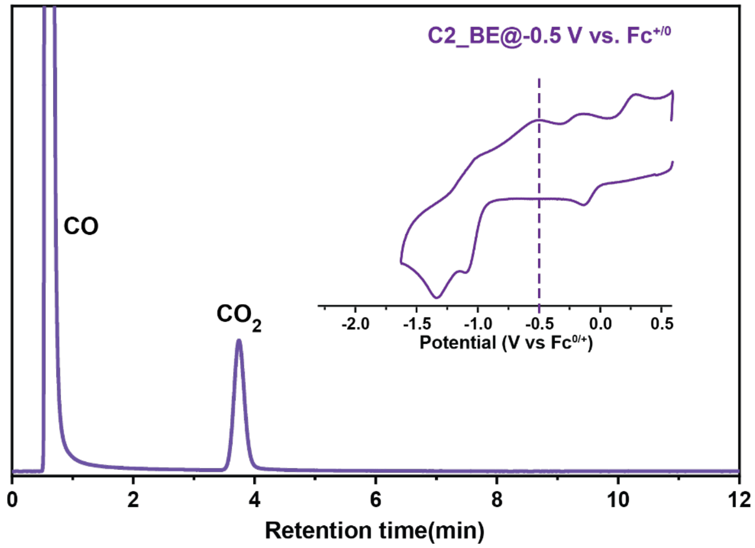

Figure S29. The gas chromatography (GC) data recorded for the head-space gas analyte generated during the bulk electrolysis of **C2** performed at -0.50 V vs. $Fc^{+/0}$ in DMF under 1 atm CO. The inset includes the cyclic voltammogram of **C2** indicating the precise position of the applied potential during the bulk electrolysis.

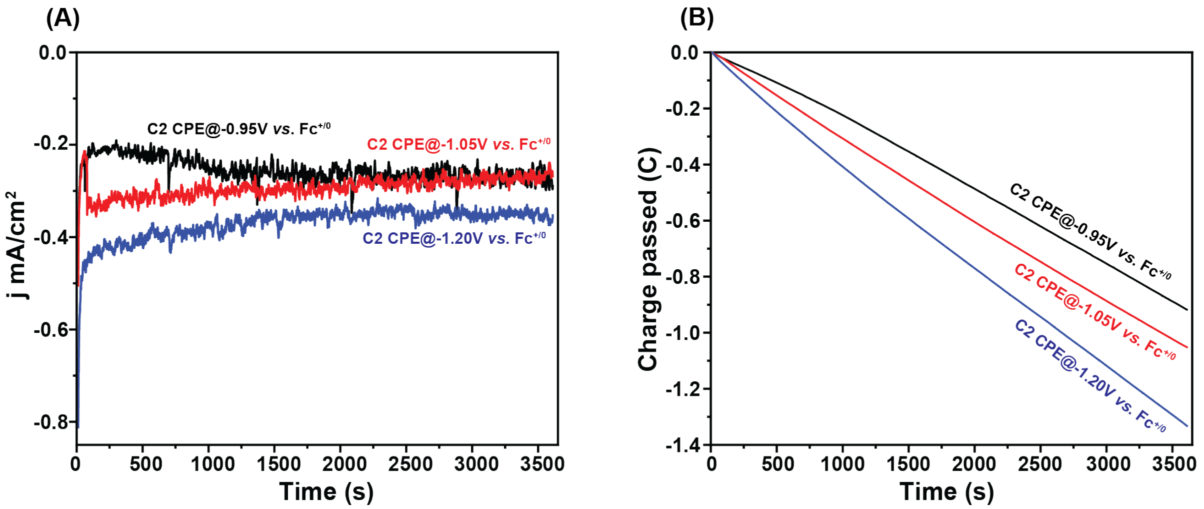

Figure S30. Bulk electrolysis of **C2** complex in CO_2 atmosphere. **(A)** Current *vs*. time plots at variable potentials, and **(B)** Corresponding charge passed *vs*. time plots at variable applied potential values.

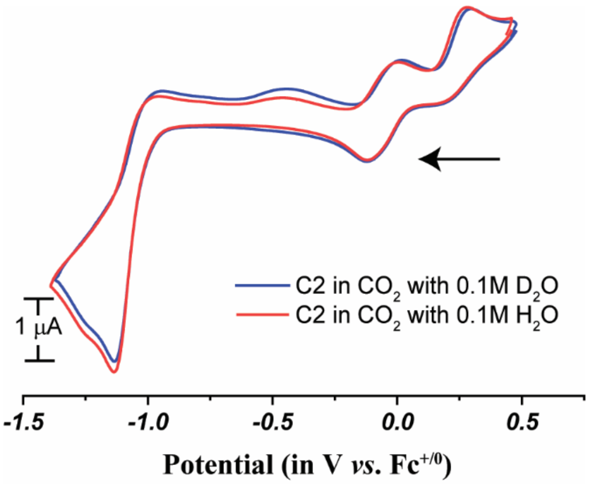

Figure S31. The comparative cyclic voltammograms of **C2** under 1 atm CO_2 in DMF in presence of H_2O and D_2O. The dark blue trace displays the data collected in the presence of 0.1 M D_2O under 1 atm CO_2 in DMF and the red trace displays the data collected in the presence of 0.1 M H_2O under 1 atm CO_2 in DMF. All data were collected at 0.05 V/s scan rate at room tempurature. The horizontal arrows depict the initial scan direction.

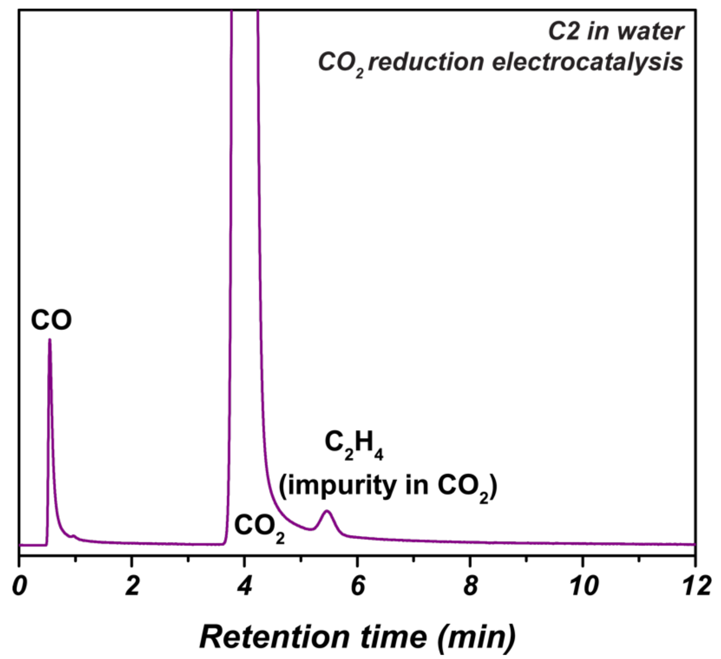

Figure S32. The gas chromatography (GC) data recorded for the head-space gas analyte generated during the bulk electrolysis of **C2** performed at -0.65 V vs. RHE in aqueous media (pH 6.5) under 1 atm CO_2.

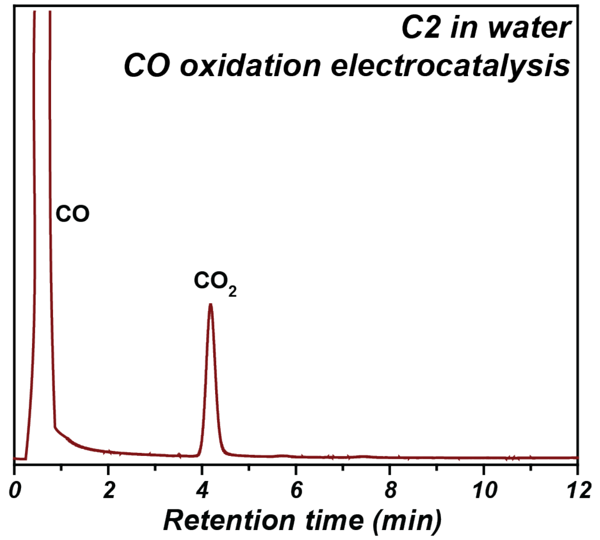

Figure S33. The gas chromatography (GC) data recorded for the head-space gas analyte generated during the bulk electrolysis of **C2** performed at -0.45 V vs. RHE in aqueous media (pH 6.5) under 1 atm CO.

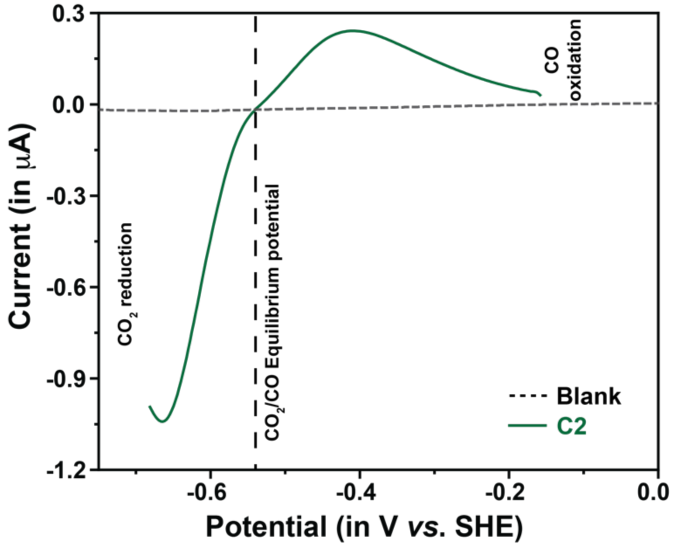

Figure S34. The background corrected data for **C2** recorded in 1:1 CO/CO_2 atmosphere in aqueous solution (pH 6.5). Scan rate of the experiment was 100 mVs^{-1}.

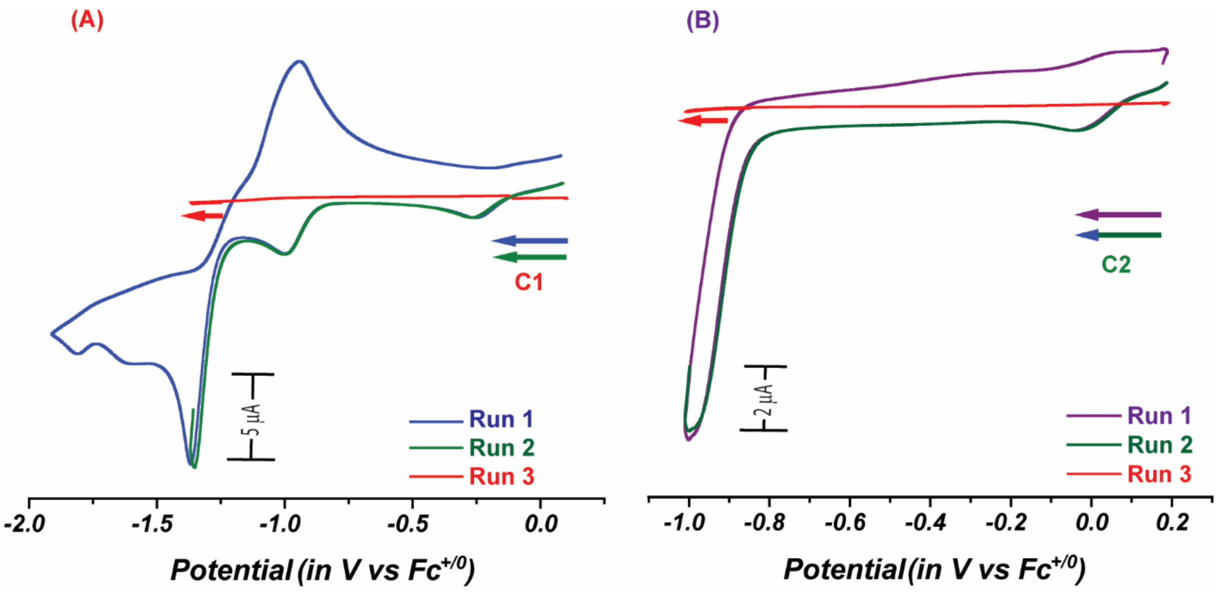

Figure S35. Rinse test experiment for (**A**) **C1** and (**B**) **C2** in DMF media. Initially a complete cyclic voltammogram (Run 1) was recorded for the complexes under saturated CO_2 atmosphere. Then working electrode was thoroughly rinsed with water followed by through polishing with 0.25 μm alumina and another round of washing. Next, Run 2 was executed on the same solution by starting the scan form the same initial position as Run 1; however, it was stopped at the potential where the maximum catalytic response was noticed. Then the working electrode was only rinsed (no polishing) with water. This electrode was then included in a blank solution (that replicates the

sample solution in all aspects other than the presence of the copper complex) and another voltammogram was recorded (Run 3) while starting from the potential where the maximum current position (catalytic CO_2 reduction) was observed. The horizontal arrows indicate the respective scan directions for each run. All data were recorded at 0.1 V/sec scan rate and 298 K.

Before Bulk Electrolysis

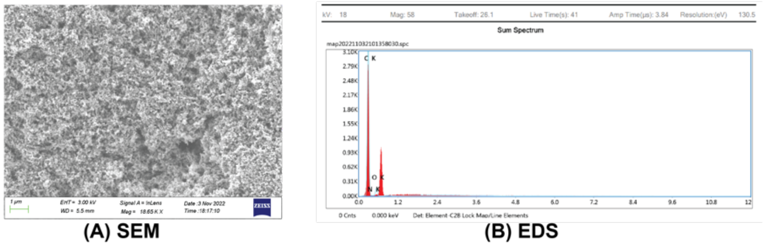

(A) SEM (B) EDS

Element	Weight %	Atomic %
C	84.51	87.61
N	0.04	0.03
O	15.81	12.36

(C) EDS Analysis

After Bulk Electrolysis

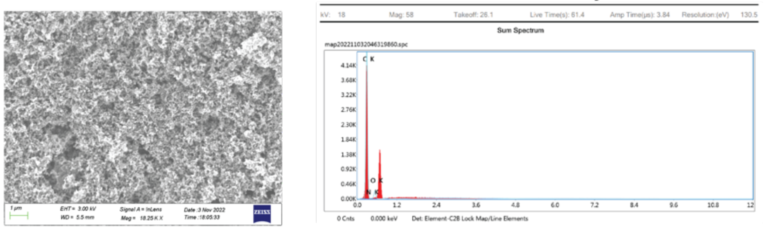

(D) SEM (E) EDS

Element	Weight %	Atomic %
C	84.53	87.84
N	0.81	0.72
O	14.65	11.43

(F) EDS Analysis

Figure S36. **(A)** The FE-SEM image, **(B)** EDX spectra, and **(C)** EDS analysis for the carbon paper working electrode *before bulk electrolysis*. **(D)** The FE-SEM image, **(E)** EDX spectra, and **(F)** EDS analysis for the carbon paper working electrode *after bulk electrolysis* of **C1** in organic medium. The Bulk electrolysis was recorded at -1.45 V vs. $FeCp_2^{+/0}$ for 3600 sec using the carbon paper working electrode, coiled Pt-wire counter electrode, and Ag wire reference electrode. The active size of the working electrode was 1 cm x 0.5 cm. Data were recorded at room temperature.

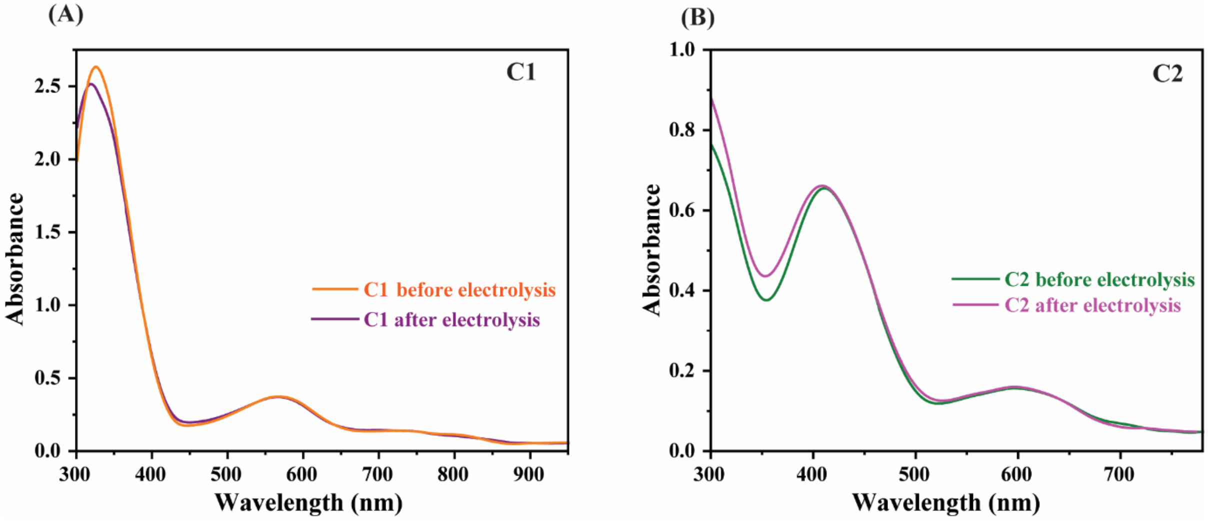

Figure S37. Optical spectra of pre and post electrolyzed solution for; (A) **C1**, orange trace- **C1** in DMF before electrolysis, violet trace- **C1** in DMF after electrolysis; (B) **C2**, orange trace- **C2** in DMF before electrolysis, violet trace- **C2** in DMF after electrolysis. All Electrolysis were recorded at -1.45 V vs. $FeCp_2^{+/0}$ for 3600 sec using the carbon paper working electrode, coiled Pt-wire counter electrode, and Ag wire reference electrode. Data was recorded at room temperature.

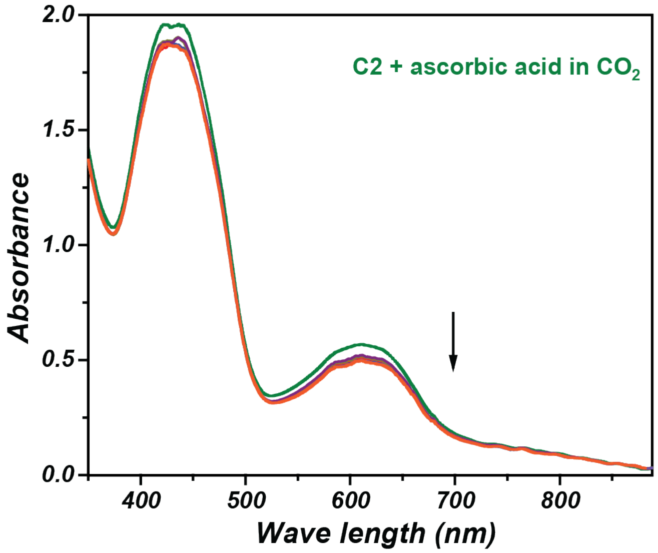

Figure S38. The gradual change in the optical spectra of **C2** following the addition of CO_2, which was reverted back with the addition of ascorbic acid.

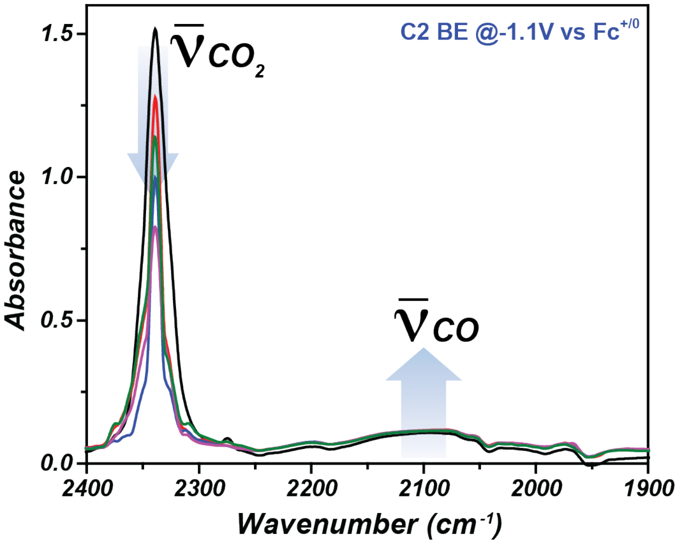

Figure S39. The sequential change in the FTIR spectra of **C2** under CO_2 atmosphere in DMF media during a bulk electrolysis experiment when -1.1 V (vs. $Fc^{+/0}$) potential was applied.

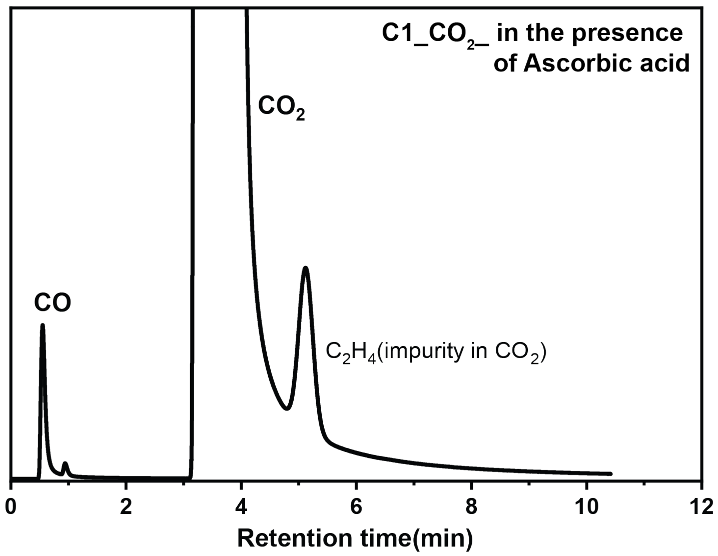

Figure S40. The GC data recorded from the chemical catalysis of **C1** performed in the presence of ten equivalents of ascorbic acid in DMF under 1 atm CO_2.

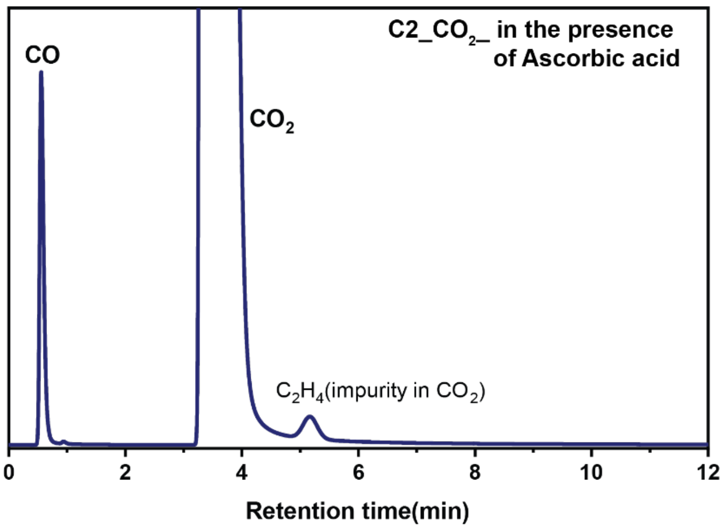

Figure S41. The GC data recorded from the chemical catalysis of **C2** performed in the presence of ten equivalents of ascorbic acid in DMF under 1 atm CO_2.

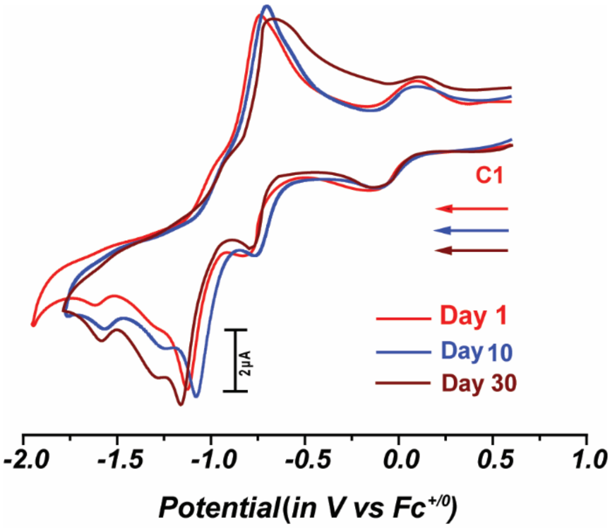

Figure S42. Comparative cyclic voltammogram data collected for a **C1** stock solution following its preparation on day 1 (red trace), day 10 (blue trace), and day 30 (brown trace). All data were recorded in 1mM DMF solution of **C1** under CO_2 atmosphere with 0.1 V/s scan rate at 289K temperature. The horizontal arrows describe the initial scan direction.

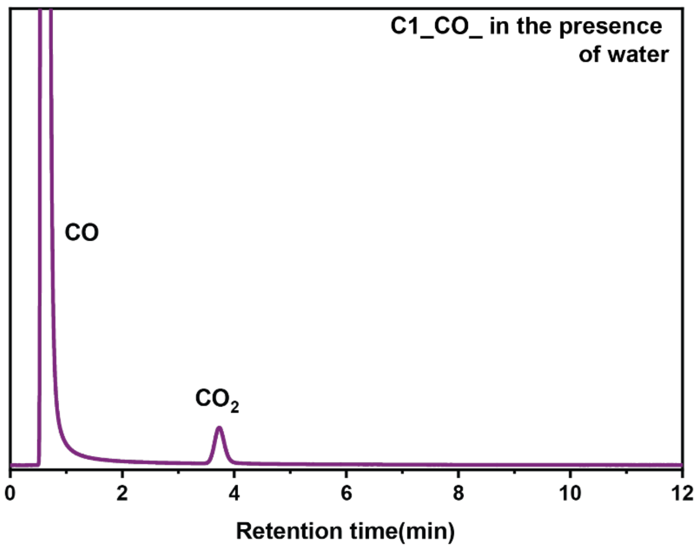

Figure S43. The GC data recorded from the chemical catalysis of **C1** performed in the presence of 6.1 M water in DMF under 1 atm CO.

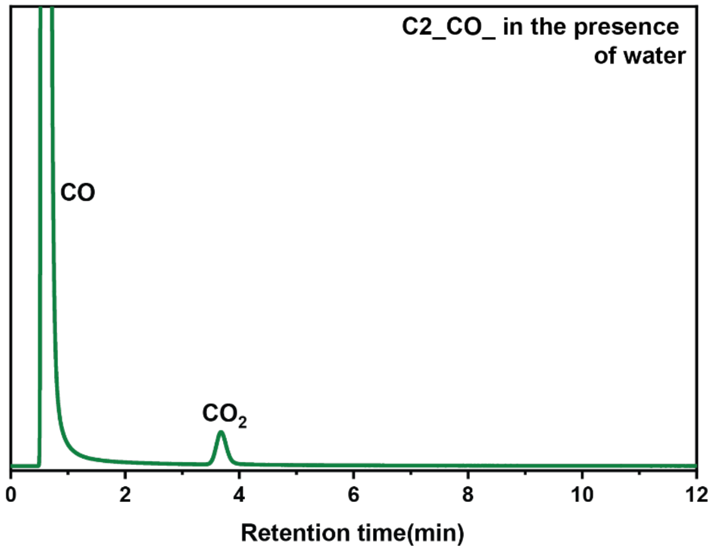

Figure S44. The GC data recorded from the chemical catalysis of **C2** performed in the presence of 6.1 M water in DMF under 1 atm CO.

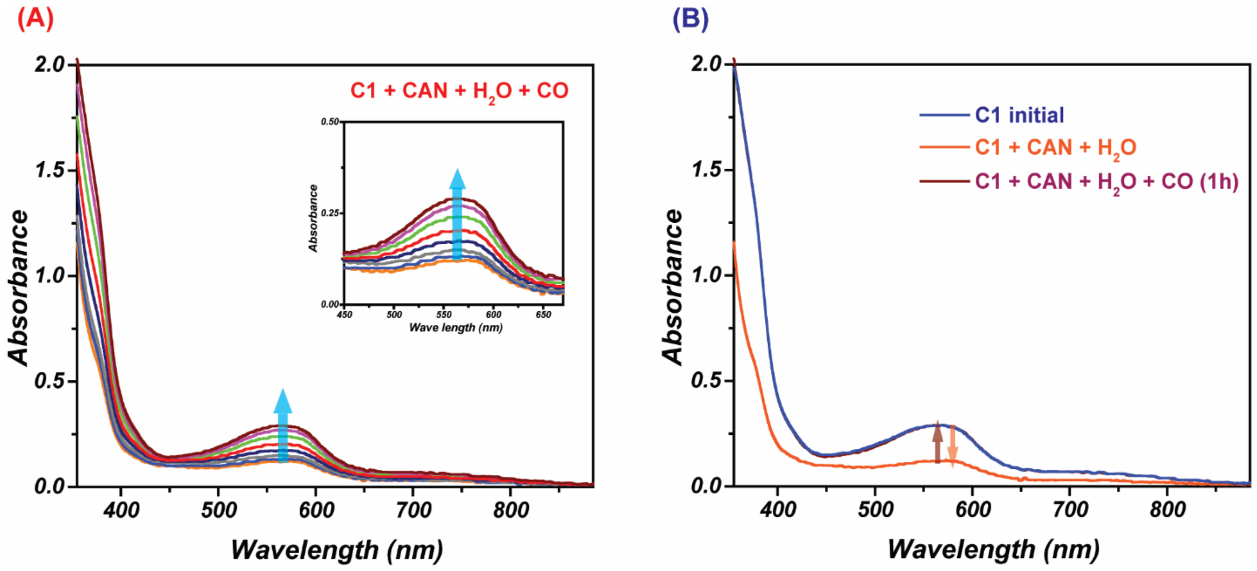

Figure S45. Optical spectra changes during CO oxidation in Cu(II) sample of **C1**. **(A)** Continuous CO purging to the Cu(II) sample of **C1** in presence of 6.1 M H_2O. **(B)** Cu(II) generation by CAN and Cu(I) formation by CO in presence of H_2O.

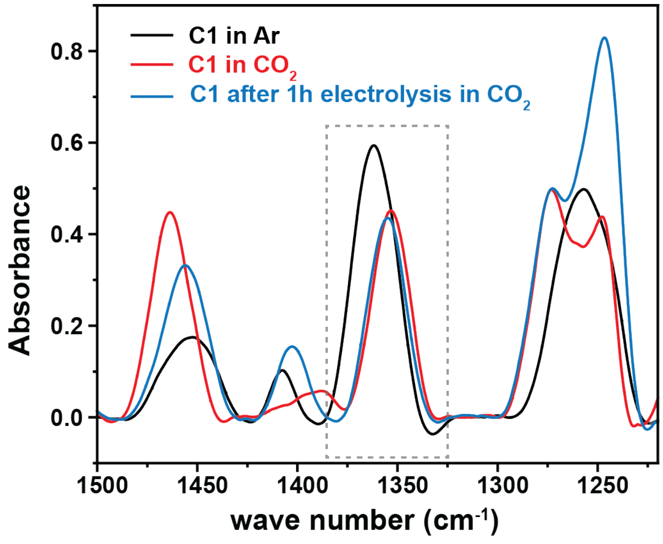

Figure S46. Comparative FTIR spectra recorded for **C1** under 100% CO_2 atmosphere during a spectroelectrochemical experiment.

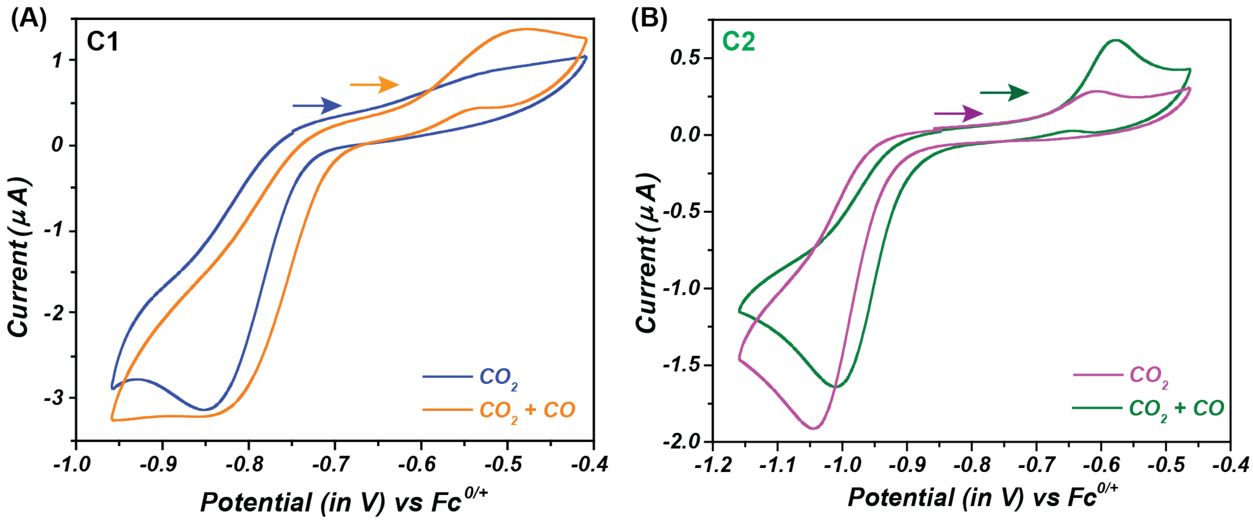

Figure S47. (A) Comparative cyclic voltammogram data recorded for **C1** under CO_2 (blue trace) and CO_2 /CO mixed atmosphere (orange trace) in the presence of 6.1 M water in DMF. **(B)** Comparative cyclic voltammogram data recorded for **C2** under CO_2 (violet trace) and CO_2 /CO mixed atmosphere (green trace) in the presence of 6.1 M water in DMF. The horizontal arrows depict the initial scan directions, respectively. All data were recorded at 298K temperature.

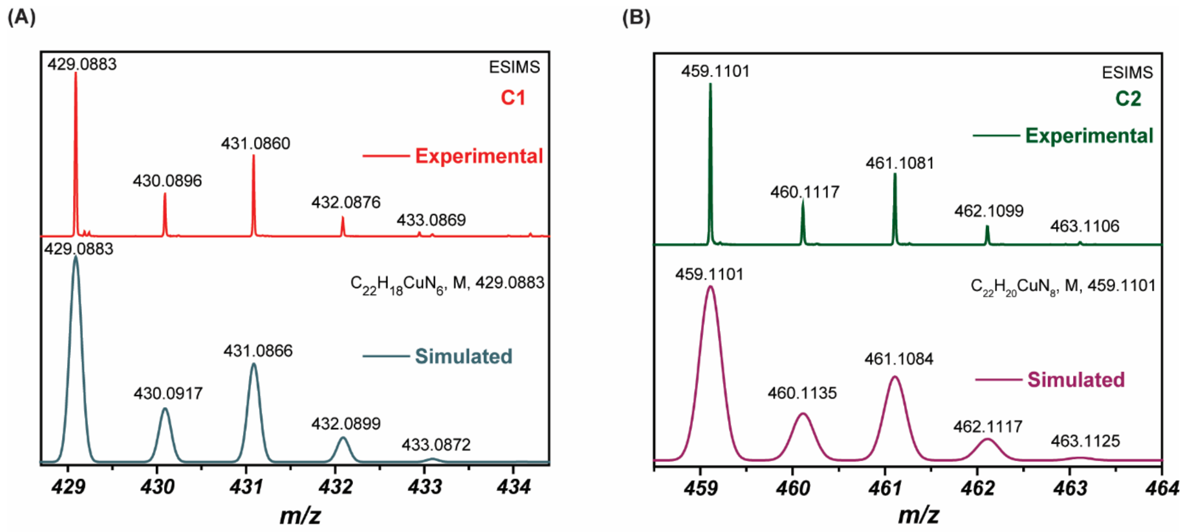

Figure S48. HRMS data along with their theoretical isotropic pattern for (**A**) **C1** and (**B**) **C2** recorded in methanol.

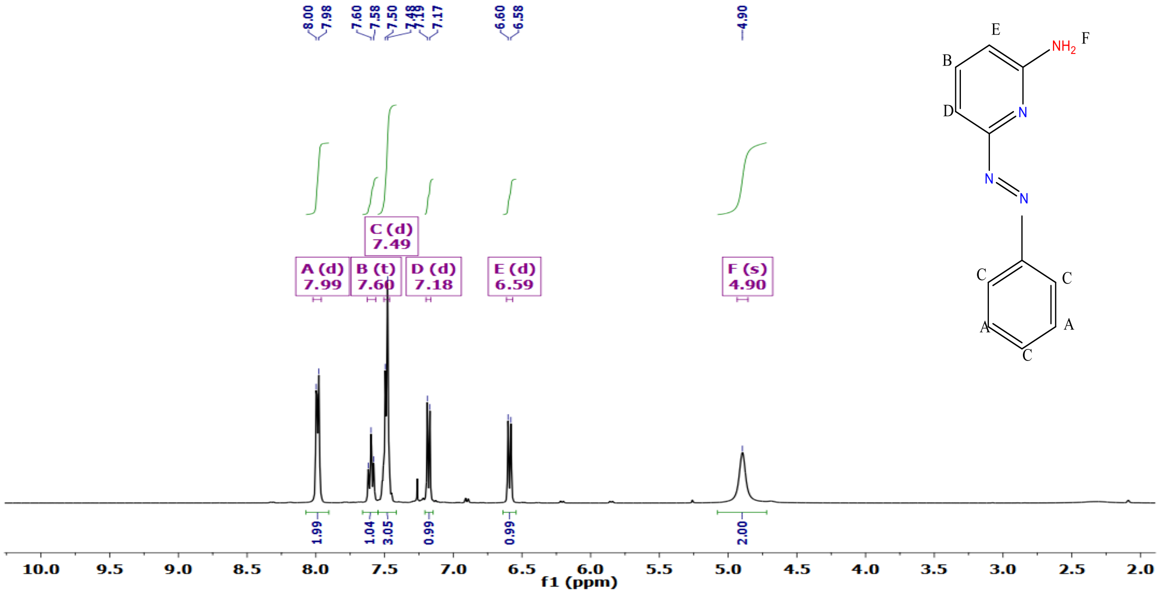

Figure S49. ^{1}H NMR spectrum of **L2** recorded in $CDCl_3$ (400 MHz, 298K).

Table S1. Crystal data and refinement details of compound **C1** and **C2**.

Identification code	**C1.** ClO_4	**C2.** Br
Empirical formula	$C_{22}H_{18}CuN_6$. ClO_4	$C_{22}H_{20}CuN_8$ Br
Formula weight	529.41	539.91
Temperature/K	150.0	100.00
Colour	Violet	Green
Crystal system	Triclinic	Monoclinic
Space group	P-1	C2/c
a/Å	9.6658(10)	15.5689(12)
b/Å	10.7152(12)	13.8237(12)
c/Å	11.9420(12)	21.1643(16)
α/°	98.669(3)	90

β/°	102.962(3)	98.376(2)
γ/°	91.602(3)	90
Volume/Å^3	1189.1(2)	4506.4(6)
Z	2	8
ρ_{calc}g/cm^3	1.479	1.592
μ/mm^{-1}	1.071	2.770
F(000)	540.0	2176.0
Crystal size/mm^3	0.21 × 0.13 × 0.05	0.211 × 0.163 × 0.11
Radiation	MoKα (λ = 0.71073)	MoKα (λ = 0.71073)
2Θ range for data collection/°	4.334 to 56.76	4.238 to 56.766
Index ranges	-12 ≤ h ≤ 12, -14 ≤ k ≤ 14, -15 ≤ l ≤ 15	-20 ≤ h ≤ 17, -18 ≤ k ≤ 18, -27 ≤ l ≤ 28
Reflections collected	60108	58090
Independent reflections	5917 [R_{int} = 0.1319, R_{sigma} = 0.0796]	5626 [R_{int} = 0.0532, R_{sigma} = 0.0286]
Data/restraints/parameters	5917/0/307	5626/0/290
Goodness-of-fit on F^2	1.024	1.106
Final R indexes [I>=2σ (I)]	R_1 = 0.0792, wR_2 = 0.1986	R_1 = 0.0411, wR_2 = 0.0963
Final R indexes [all data]	R_1 = 0.1470, wR_2 = 0.2436	R_1 = 0.0514, wR_2 = 0.1003
Largest diff. peak/hole / e Å^{-3}	3.60/-1.03	1.32/-0.70
CCDC	-	2122626

References

1. Das, D. *et al.* Oxidation State Analysis of a Four-Component Redox Series [Os(pap)2(Q)]n Involving Two Different Non-Innocent Ligands on a Redox-Active Transition Metal. *Inorg. Chem.* **50**, 7090–7098 (2011).

2. Datta, D. & Chakravorty, A. Bis(2-(phenylazo)pyridine)copper(I) and -copper(II): ligand .pi. acidity and high formal potential of the copper(II)-copper(I) couple. *Inorg. Chem.* **22**, 1085–1090 (1983).
3. Sheldrick, G. M. *SADABS, Version 2.05. A Software for Empirical Absorption Correction.* (2002).
4. Sheldrick, G. M. Crystal structure refinement with SHELXL. *Acta Crystallogr. Sect. C Struct. Chem.* **71**, 3–8 (2015).
5. Dolomanov, O. V., Bourhis, L. J., Gildea, R. J., Howard, J. a. K. & Puschmann, H. OLEX2: a complete structure solution, refinement and analysis program. *J. Appl. Crystallogr.* **42**, 339–341 (2009).
6. Sheldrick, G. M. A short history of SHELX. *Acta Crystallogr. A* **64**, 112–122 (2008).
7. Macrae, C. F. *et al.* Mercury 4.0: from visualization to analysis, design and prediction. *J. Appl. Crystallogr.* **53**, 226–235 (2020).
8. Spek, A. L. Single-crystal structure validation with the program PLATON. *J. Appl. Crystallogr.* **36**, 7–13 (2003).
9. Westrip, S. P. publCIF: software for editing, validating and formatting crystallographic information files. *J. Appl. Crystallogr.* **43**, 920–925 (2010).
